# Ferroptosis inhibitor improves outcome after early and delayed treatment in mild spinal cord injury

**DOI:** 10.1007/s00401-024-02758-2

**Published:** 2024-06-22

**Authors:** Fari Ryan, Christian Blex, The Dung Ngo, Marcel A. Kopp, Bernhard Michalke, Vivek Venkataramani, Laura Curran, Jan M. Schwab, Klemens Ruprecht, Carolin Otto, Priya Jhelum, Antje Kroner, Samuel David

**Affiliations:** 1 Centre for Research in Neuroscience, The BRAiN Program, The Research Institute of the McGill University Health Centre, Livingston Hall, Room L7-210, 1650 Cedar Ave., Montreal, QC H3G 1A4 Canada; 2 Clinical and Experimental Spinal Cord Injury Research (Neuroparaplegiology), Department of Neurology and Experimental Neurology, Charité - Universitätsmedizin Berlin, Berlin, Germany; 3 Research Unit Analytical BioGeoChemistry, Helmholz Zentrum München, German Research Center for Environmental Health, Neuherberg, Germany; 4 Comprehensive Cancer Center Mainfranken, University Hospital Würzburg, 97080 Würzburg, Germany; 5 Belford Center for Spinal Cord Injury and Departments of Neurology and Neurosciences, The Ohio State University, Columbus, OH 43210 USA; 6 Department of Neurology, Charité-Universitätsmedizin Berlin, Corporate Member of Freie Universität Berlin and Humboldt-Universität zu Berlin, Berlin, Germany; 7 Department of Neurosurgery, Medical College of Wisconsin, Milwaukee, WI 53226 USA

**Keywords:** Iron toxicity, Spinal cord injury, Antioxidants, NCOA4, Ferritin, Lipid peroxidation

## Abstract

We show that redox active iron can induce a regulated form of non-apoptotic cell death and tissue damage called ferroptosis that can contribute to secondary damage and functional loss in the acute and chronic periods after spinal cord injury (SCI) in young, adult, female mice. Phagocytosis of red blood cells at sites of hemorrhage is the main source of iron derived from hemoglobin after SCI. Expression of hemeoxygenase-1 that induces release of iron from heme, is increased in spinal cord macrophages 7 days after injury. While iron is stored safely in ferritin in the injured spinal cord, it can, however, be released by NCOA4-mediated shuttling of ferritin to autophagosomes for degradation (ferritinophagy). This leads to the release of redox active iron that can cause free radical damage. Expression of NCOA4 is increased after SCI, mainly in macrophages. Increase in the ratio of redox active ferrous (Fe^2+^) to ferric iron (Fe^3+^) is also detected after SCI by capillary electrophoresis inductively coupled mass spectrometry. These changes are accompanied by other hallmarks of ferroptosis, i.e., deficiency in various elements of the antioxidant glutathione (GSH) pathway. We also detect increases in enzymes that repair membrane lipids (ACSL4 and LPCAT3) and thus promote on-going ferroptosis. These changes are associated with increased levels of 4-hydroxynonenal (4-HNE), a toxic lipid peroxidation product. Mice with mild SCI (30 kdyne force) treated with the ferroptosis inhibitor (UAMC-3203-HCL) either early or delayed times after injury showed improvement in locomotor recovery and secondary damage. Cerebrospinal fluid and serum samples from human SCI cases show evidence of increased iron storage (ferritin), and other iron related molecules, and reduction in GSH. Collectively, these data suggest that ferroptosis contributes to secondary damage after SCI and highlights the possible use of ferroptosis inhibitors to treat SCI.

**Supplementary Information:**

The online version contains supplementary material available at 10.1007/s00401-024-02758-2.

## Introduction

Spinal cord injury (SCI) results in marked accumulation of iron at the injury site that can arise from three main sources: (i) iron from hemoglobin in red blood cells (RBCs) found at the site of hemorrhage, (ii) iron stores (in ferritin and heme-containing molecules) released from damaged CNS cells that are phagocytosed by macrophages; (iii) dysregulation of cellular iron homoeostasis mechanisms [8, 42]. RBCs are a rich source of iron, as each RBC contains about 240 million molecules of hemoglobin and each molecule of hemoglobin has 4 heme groups, each with an atom of iron at its center. Therefore, each RBC has over a billion atoms of iron. Phagocytosis of RBCs, therefore, results in tremendous iron load in macrophages at the site of SCI. Iron released from heme via hemeoxygenase-1 (HO-1) from these sources is stored safely in ferritin [1]. Iron exists in either the redox active Fe^2+^ (ferrous) form or the redox inactive Fe^3+^ (ferric) form. Ferritin is composed of 24 heavy (FTH) and light (FTL) subunits which join to form a hollow sphere that holds up to 4500 atoms of iron [1]. The release of iron stored in ferritin occurs either by a process of degradation in autophagosomes called ferritinophagy [31] or due to loss of viability of the macrophages/microglia themselves at late stages of pathology leading to the release of redox active iron [8]. We have previously shown evidence of the latter process several weeks after SCI, when lipid peroxidation is seen in iron containing macrophages in the core of the lesion [42]. This suggests that iron released from macrophages could trigger oxidative damage in the chronic period after SCI.

Iron chelator treatments have had limited success in models of SCI. This may be in part due to difficulty in chelating intracellular iron and clearing the chelator-iron complex out of the injured CNS. An exciting development in the iron field came in 2012, with the discovery of a novel form of iron-mediated cell death called ferroptosis [10]. Ferroptosis is induced by iron-mediated free radicals triggering lipid peroxidation in the absence of sufficient levels of antioxidant (glutathione) defense [46, 52]. This results in the generation of damaging, toxic lipid radicals. Damage to lipids is particularly problematic in the lipid rich environment of the CNS, as it can result in myelin damage and neurodegeneration as occurs in SCI [19, 21, 33, 34, 42]. Ferroptosis has been shown to occur in several neurological disorders [8], and some recent studies suggest evidence for ferroptosis in SCI [8].

In this study we characterized the expression of various ferroptosis markers that include those involved in iron accumulation, iron release, deficient glutathione metabolism, and increased lipid peroxidation at different times after spinal cord contusion injury in mice. Most of the iron in SCI is derived from RBC phagocytosis and stored in ferritin. We demonstrate increased expression of nuclear receptor coactivator 4 (NCOA4), a key molecule that transports ferritin to autophagosomes for degradation and release of redox active iron. We also detected increased redox active Fe^2+^ iron and lipid peroxidation at acute and chronic stages after SCI. Changes in levels of ferroptosis markers were also detected in CSF and serum of SCI patients. Treatment of mice with mild SCI with the ferroptosis inhibitor, UAMC-3203, during either the early (1–14 days) or late (28–42 days) periods after SCI improved locomotor recovery and secondary damage.

## Materials and methods

### Spinal cord injury and treatment with ferroptosis inhibitor

Female C57BL/6 mice (8 weeks of age) were used. Mice were deeply anesthetized with a mixture of ketamine, xylazine, and acepromazine (50:5:1 mg/kg), a partial laminectomy done at vertebral level T11. A mild force contusion injury (30 kilo dyne force, 200–400 µm displacement) or moderate force contusion injury (40 kilo dyne force, 300–500 µm spinal cord displacement) was made using the Infinite Horizons impactor (Precision Scientific Instrumentation) [18, 19]. Locomotor recovery was assessed using the 9-point Basso mouse scale (BMS) [2] carried out by two independent observers trained in the Basso laboratory at Ohio State University, and the consensus scores recorded. These analyses were performed by observers blinded to experimental groups. All procedures were approved by the McGill University Animal Care Committee and followed the guidelines of the Canadian Council on Animal Care. All animal work adhered to the ARRIVE guidelines [40]. Mice were fed a standard mouse chow and water ad libitum and provided with environmental enrichment. They were housed in a specific pathogen-free facility in individually ventilated cages in a temperature and humidity-controlled environment at 21.5 °C on a 12 h light/dark cycle.

Mice were assigned randomly to treatment and control groups. Mice in the treatment group received the ferroptosis inhibitor UAMC-3203-HCL [9] (15 mg/kg body weight; HY-112909A-100MG, MedChem Express) solubilized in 3% DMSO in normal saline, and injected intraperitoneally, daily for either early (acute) (1–14 days) or delayed (28–42 days) treatment groups after SCI. Mice in the control group received the same volume of vehicle (3% DMSO in normal saline) intraperitoneally for the same duration. The number of mice used for each group is indicated in text in the Results section and in the figure legends. Sample size was calculated based on our historical experience with SCI for medium effect size (f = 0.25), a confidence coefficient of 0.05 and a statistical power of 0.80. The individuals doing the analyses were blinded to the treatment and control groups.

### Western blotting

Mice were perfused intracardially with PBS under deep anesthesia, and approximately 5 mm of the injured spinal cords centered on the lesion epicenter was removed as we have done previously [28, 33] at various times after SCI as indicated, and equivalent regions taken from uninjured (naïve) mice for baseline evaluations. Protein was extracted from these samples with 1% Nonidet P-40 (Sigma), 1% sodium deoxycholate (BDH Chemicals), 2% SDS, 0.15 M sodium phosphate (pH 7.2), and 2 mM EDTA, containing a mixture of protease inhibitors (Roche Diagnostics) as described previously [24]. Protein concentrations were estimated using the Bio-Rad DC protein assay following manufacturer’s instructions (Cat. No 500-0121). 25 µg of protein per sample were separated by sodium dodecyl sulfate polyacrylamide electrophoresis (SDS–PAGE) gels 4–12% (Novex, Life Technologies), transferred to polyvinylidene fluoride membrane (PVDF) (Millipore, Billerica, MA). Membranes were blocked in 5% milk in 0.05% PBS Tween-20 and incubated overnight at 4 °C with one of the following primary antibodies: rabbit anti-ferritin (1:500; Sigma-Aldrich Inc.; F5012), rabbit anti-DMT1 (1:1000; Alpha Diagnostics: NRAMP23-S), mouse anti-TfR1 (1:1000; Invitrogen; 13–6800), mouse anti-NCOA4 (1:500; Abnova; H00008031-M04), rabbit anti-LPCAT3 (1:500; Novus Biologicals; NBP3-04752), rabbit anti-ACSL4 (1:1000; Abcam; ab155282), rabbit anti-HO-1 (1:1000; Abcam; ab68477), rabbit anti-xCT (1:1000; Abcam; ab175186), mouse anti-GPX4 (1:500; Alpha Diagnostics; MAB5457), rabbit anti-4-HNE, (1:500; Abcam; ab46545). Blots were washed and incubated with horseradish peroxidase-conjugated IgG (1:10,000–100,000; Jackson ImmunoResearch, West Grove, PA, United States) and visualized with enhanced chemiluminescence. Equal loading of proteins was estimated by re-probing the membranes with mouse anti-GAPDH antibodies (1:1000; EMD Millipore, MAB374). The blots were quantified using ImageJ/Fiji software (version2.3.0/1.53q, National Institutes of Health).

### Immunofluorescence and confocal microscopy of mouse tissue

Under deep anesthesia, mice were transcardially perfused with phosphate buffer followed by 4% paraformaldehyde in 0.1 M phosphate buffer. A 5 mm length of spinal cord was removed, centered on the epicenter of injury, postfixed for 1 h, cryoprotected in 30% sucrose in 0.1 M PB for 48 h, and serial 14 µm thick cryostat sections cut and picked-up on glass slides. Tissue sections were incubated with 0.3% Triton X-100 (Sigma-Aldrich), 5% normal goat serum (Jackson ImmunoResearch Inc.), 2% ovalbumin (Sigma-Aldrich) in phosphate buffered saline for 3 h at room temperature (RT) to block non-specific binding of antibodies. Sections were then incubated overnight at 4ºC with one of the following primary antibodies against: rabbit anti-ferritin (1:200; Sigma-Aldrich Inc.; F5012), rabbit anti-NCOA4 (1:400; Invitrogen; PA5-96398), rabbit anti-4-HNE, 1:100; Abcam; ab46545), rat anti serotonin (1:100; Abcam; ab6336), and double labeled with either rat anti-CD11b (for macrophages/microglia; 1:200; AbD Serotec; MCA711), rat anti-glial fibrillary acidic protein (GFAP) (for astrocytes; 1:500; Invitrogen; 13-0300), rabbit anti-GFAP (for astrocytes; 1:500; Agilent Dako; Z0334), guinea pig anti-NeuN (for neurons; 1:400; Synaptic Systems; 266004), mouse anti-CC1 (for oligodendrocytes; 1:200; EMD Millipore; OP80), or mouse anti-neurofilament-200 (for axons; 1:300; Sigma-Aldrich Inc; N0142). Sections were washed in 0.05% PBS-Tween-20 and incubated with appropriate fluorescent conjugated secondary antibodies (goat anti-rabbit Alexa Fluor 488, goat anti-rabbit Alexa Fluor 568, goat anti-rat Alexa Fluor 568, goat anti-guinea pig Alexa Fluor 568 (all 1:500, Invitrogen), or goat anti-mouse Alexa Fluor 568. Tissue sections were viewed with a confocal laser scanning microscope (FluoView FV1000, Olympus) and micrographs taken with the FV10-ASW 3.0 software (Olympus). Negative controls excluding primary antibodies were done and showed no staining. For comparing between groups, the same setting was applied in all images for each immunostaining. For quantification of immunofluorescence data, area of fluorescence and fluorescence intensity (as measured by Integrated Density [IntDen], which is the product of area and mean gray value) were quantified with ImageJ, and the graphs were plotted using Graphpad prism.

### Histology and quantification

For analysis of myelin loss, lesion size, and serotonergic (5-HT) innervation, serial cryostat sections of mouse SCI tissue were taken at the end stage and picked up on glass slides. (1) To assess myelin sparing, tissue sections were stained with Luxol Fast Blue (LFB) as described [25]. For this, sections were first dehydrated by immersing in graded ethanol solutions for 2 min each (50–95%) and then placed in a 0.1% LFB solution overnight at 37 °C. Next day, after cooling the slides at 4 °C for 1 h, they were dipped in 95% ethanol, and then in dH_2_O, and incubated in 0.05% lithium carbonate solution for 5 min. After placing the slides in dH_2_O for 1 min, they were dehydrated in graded ethanol solutions (70–100%), placed in xylene 3 times for 5 min, mounted and cover-slipped. Images of the dorsal region of the spinal cords were taken using Zeiss AxioScan.Z1 slide scanner using ZEN image software (Carl Zeiss, Germany). For quantification, the threshold feature of ImageJ was used to measure the area of spared myelin in the region of the dorsal white matter. The ratio of spared myelin was measured at different intervals over a 2 mm length of the cord. Data is expressed as the percentage and graphs plotted using GraphPad prism. (2) To assess lesion size, serial cryostat sections were stained by immunofluorescence labeling with anti-GFAP (astrocyte labeling) using the protocol described above in the “Immunofluorescence” section. Using ImageJ software, the lesion size was quantified as a percentage of the total cross-sectional area of the spinal cord, presenting the data as the area of GFAP loss relative to the entire spinal cord area. (3) To assess serotonergic (5-HT) sprouting, cryostat sections from 1 mm caudal to lesion epicenter were blocked and incubated with rat monoclonal anti-5-HT (1:100; Abcam, ab6336) antibody and appropriate secondary antibody (goat anti-rat Alexa Fluor 568). Fluorescence intensity as measured by Integrated Density [IntDen] of 5-HT labeling of the ventral horn 1 mm caudal to the lesion epicenter was measured separately in the right and left ventral horns using the ImageJ software as described previously [19]. Subsequently, the obtained values for each animal were averaged.

### Capillary electrophoresis-inductively coupled mass spectrometry (CE-ICP-MS)

Spinal cord from normal uninjured (naïve) and after 24 h, 7d, 14 d, and 5 weeks after SCI were collected and lysed in modified RIPA lysis buffer containing 1 × PBS pH 7.4, 0.5% sodium deoxycholate, 1% NP-40 on ice using a sonicator. Cell lysates were centrifuged at 14,000 RPM for 20 min, and one aliquot of supernatants were sent on dry ice to the Helmholtz Center Munich, Germany. The remaining aliquot was used for protein assay using the Bio-Rad DC kit (Cat. No 500-0121) and to estimate the amount of ferritin using the mouse ferritin ELISA kit from Abcam (ab157713). Speciation and quantification of Fe^2+^, Fe^3+^, Fe-load of ferritin, and total iron was performed by capillary electrophoresis inductively coupled plasma mass spectrometry (CE-ICP-DRC-MS) as we previously described [37, 38]. In brief, samples were analyzed on a "PrinCe 706″ CE system equipped with an uncoated capillary (85 cm × 50 µm ID) and a laboratory-constructed CE-ICP-MS interface for element selective quantification of separated iron redox species at ICP-DRC (dynamic reaction cell)-MS. DRC technology with NH_3_ as reaction gas was employed for spectral interference-free detection of the ^56^Fe isotope. Fe^2+^/Fe^3+^ separation and quantification were performed in 20 mM HCl-electrolyte at + 25 kV separation voltage and ^56^Fe isotope detection at ICP-DRC-MS. For quality control total iron was additionally determined by ICP-sf-MS and values compared to the sum of iron species quantified by CE-ICP-DRC-MS. Data are presented as total iron levels, Fe^2+^/Fe^3+^ ratio, and ferritin-iron content.

### GSH assay in mouse SCI

Mice were perfused with PBS via the heart, and 5 mm of the spinal cord centered on the lesion epicenter and equivalent regions from the uninjured (naïve) spinal cord was taken at different timepoints after SCI for analysis. GSH levels were measured using the Glutathione assay kit from Cayman Chemicals (Ann Arbor, MI Cat# 703002) according to the manufacturer’s instructions, as we have previously reported [25]. Briefly, tissues were homogenized with a sonicator in 50 mM MES buffer containing 1 mM EDTA, followed by centrifugation at 10,000*g* for 15 min at 4 °C. Supernatants were obtained and one aliquot used for protein assay (Bio-Rad DC kit; Cat.No #500-0121) and the other aliquot deproteinated using equal quantity of metaphosphoric acid and centrifuged at 3000*g* for 3 min followed by mixing with 4 M triethanolamine. Samples, standard, assay buffer, co-factor, enzyme mixture and Ellman’s reagent were added to 96-well plates and incubated for 30 min. Absorbance levels of each sample and standard were measured at 412 nm with a micro plate reader. Total GSH levels were determined using a standard curve calculated according to the manufacturer’s instructions and normalized to the protein concentration.

### Analysis of human CSF and serum samples for ferroptosis markers

#### Patients and ethics approval

Spinal cord injury patients were recruited for the single centre SCISSOR study at the Trauma Hospital Berlin as described in the clinical study protocol [27]. The study sample comprised n = 12 patients with motor complete SCI (ASIA impairment scale A and B) and cervical neurological level (C4 to C6) (see Table 1 for more details). The study was approved by the Berlin State Ethics Board located at the Landesamt für Gesundheit und Soziales (LaGeSo), Berlin, Germany (13/0127-EK13), and the Federal Institute for Drugs and Medical Devices (BfArM). CSF and serum samples from control patients were recruited through the CSF/serum biobank at the Central Biomaterialbank (ZeBanC) of Charité-Universitätsmedizin Berlin, which stores CSF and serum samples from patients with various neurological diseases undergoing lumbar punctures for routine diagnostic work-up. Control patients without neuroimmunological or neurodegenerative diseases, cerebral bleeding or traumatic injury of the central nervous system were selected for this study (n = 12). The Charité Ethics Board approved the collection and storage of CSF/serum samples (EA4/018/17) for scientific research and for the use of the samples for analyzing markers of ferroptosis after traumatic SCI (EA1/209/23). All studies were conducted in compliance with the Declaration of Helsinki and all patients gave their written informed consent before study participation. Ethics approval was also obtained from the Research Institute of the McGill University Health Centre (RI-MUHC), Montreal (project # 2021-6876; FERISCI).

**Table 1:** Patient characteristics of SCI and Controls

Patient number	Sex	Age	Group	Disease or symptoms
1	Female	46–60	Spinal cord injury	Spinal cord injury
2	Male	46–60		Spinal cord injury
3	Male	16–30		Spinal cord injury
4	Male	61–75		Spinal cord injury
5	Male	31–45		Spinal cord injury
6	Male	46–60		Spinal cord injury
7	Male	31–45		Spinal cord injury
8	Male	31–45		Spinal cord injury
9	Male	16–30		Spinal cord injury
10	Male	61–75		Spinal cord injury
11	Male	61–75		Spinal cord injury
12	Female	31–45		Spinal cord injury
13	Female	31–45	Control	Somatization disorder
14	Male	46–60		Somatization disorder
15	Male	31–45		Somatization disorder
16	Female	46–60		Somatization disorder
17	Male	16–30		Somatoform pain disorder
18	Male	46–60		Vertigo, tumble
19	Male	31–45		Headache
20	Male	46–60		Headache
21	Male	46–60		Headache
22	Male	61–75		Headache
23	Male	16–30		Headache
24	Male	31–45		Migraine without aura

Patient characteristics of SCI and control group patients. The age of the patients is reported in 15 years groups as suggested by DeVivo et al., 2011

DeVivo, M. J., Biering-Sorensen, F., New, P. & Chen, Y. Standardization of data analysis and reporting of results from the International Spinal Cord Injury Core Data Set. Spinal Cord 49, 596–599. https://doi.org/10.1038/sc.2010.172 (2011)

#### Preparation of samples

Serum and CSF samples were collected under sterile conditions. All samples were processed for storage as soon as possible, at the latest within 8 h of withdrawal by centrifugation at 3000*g* for 10 min. Supernatants were stored at − 80 °C, with central temperature control up to subsequent batch analysis. We acknowledge the collaboration with the Central Biobank of the Charité (ZeBanC).

#### Experimental procedures

Ferritin was quantified by ELISA (#DY3541-05, R&D Systems, Minneapolis, MN, USA) according to the manufacturer’s instructions. Concentrations were calculated using a subsequent four-parameter logistic regression based on the standard analyte concentrations using GraphPad PRISM software (Dotmatics, Boston, MA, USA).

Electrochemiluminescence immunoassays (Meso Scale Diagnostics, Rockville, MD, USA) were used to quantify the analytes Hepcidin (#F21J1), Hemopexin (#F217F), Haptoglobin (#F21YF), sTfR-1 (#F21YJ) and Haemoglobin alpha (#F217E) based on the respective R-plex antibody sets according to the protocols supplied by the manufacturer and applying the MSD DISCOVERY WORKBENCH 4.0 software.

For total glutathione quantification, CSF was prepared as described by [41] in section 1J for plasma/CSF and the enzymatic recycling method carried out as described in section 2A as ‘GSH assay method for 96-well microtiter plate assay’. Due to the low levels of glutathione present in CSF, we increased the suggested absorbance reading interval (A iv) to 5 min and repeated the readings 8-times up to a total of 40 min.

### Statistical analyses

#### Human samples

As the clinical sample analysis is of exploratory nature, all available cases from the SCISSOR study (n = 12) were used. A case number of n = 12 can be considered sufficient for an orientational evaluation (Julious SA, Sample size of 12 per group rule of thumb for a pilot study, Pharmaceutical statistics. 2005, Vol.4(4), pp. 287–291; 10.1002/pst.185). Patients of the control and comparison group were matched based on age and sex. Clinical data are presented as single data points or as median with quartiles. Statistical test for exploratory group comparison is the Mann–Whitney-U-Test. Exploratory p-values should be interpreted with caution as they were not adjusted for multiple testing. We calculated Generalized Estimating Equation (GEE) models for longitudinal CSF parameters in the SCI patient group to analyse the effects of injury severity and time course on the CSF parameters. For each GEE model the respective CSF biomarker at two time points (14 days vs. 42 days) and the ASIA impairment scale of the SCI patients (AIS A vs. AIS B) determined at baseline were included as explanatory variables. The continuous CSF parameter value as exploratory variable was log-transformed (natural logarithm) to achieve a gaussian-like distribution. The effect estimates were reported with 95% confidence interval (CI). The statistical significance level was set to *p* = 0.05. Statistical analyses were carried out using R version 4.0.0. and the package geepack (Halekoh U, Højsgaard S, Yan J (2006). “The R Package geepack for Generalized Estimating Equations.” *Journal of Statistical Software*, 15/2, 1–11).

#### Mouse samples

Data are shown as Mean ± Standard Error of the Mean (SEM) or Standard Deviation (SD). Statistical tests were performed using GraphPad Prisms 9.4.1 software. Statistical analyses were performed by using Mann–Whitney-U test, one-way or two-way repeated-measures (RM)-ANOVA with post hoc Tukey or Bonferroni test for multiple comparisons as indicated. Differences were considered significant at *p* < 0.05.

## Results

Changes in various ferroptosis markers and iron homeostasis proteins were assessed in the spinal cord after moderate (40 kdyne) force injury.

### Increased expression of molecules involved in iron mobilization, uptake, storage and release in the injured spinal cord

**Fig. 1:**
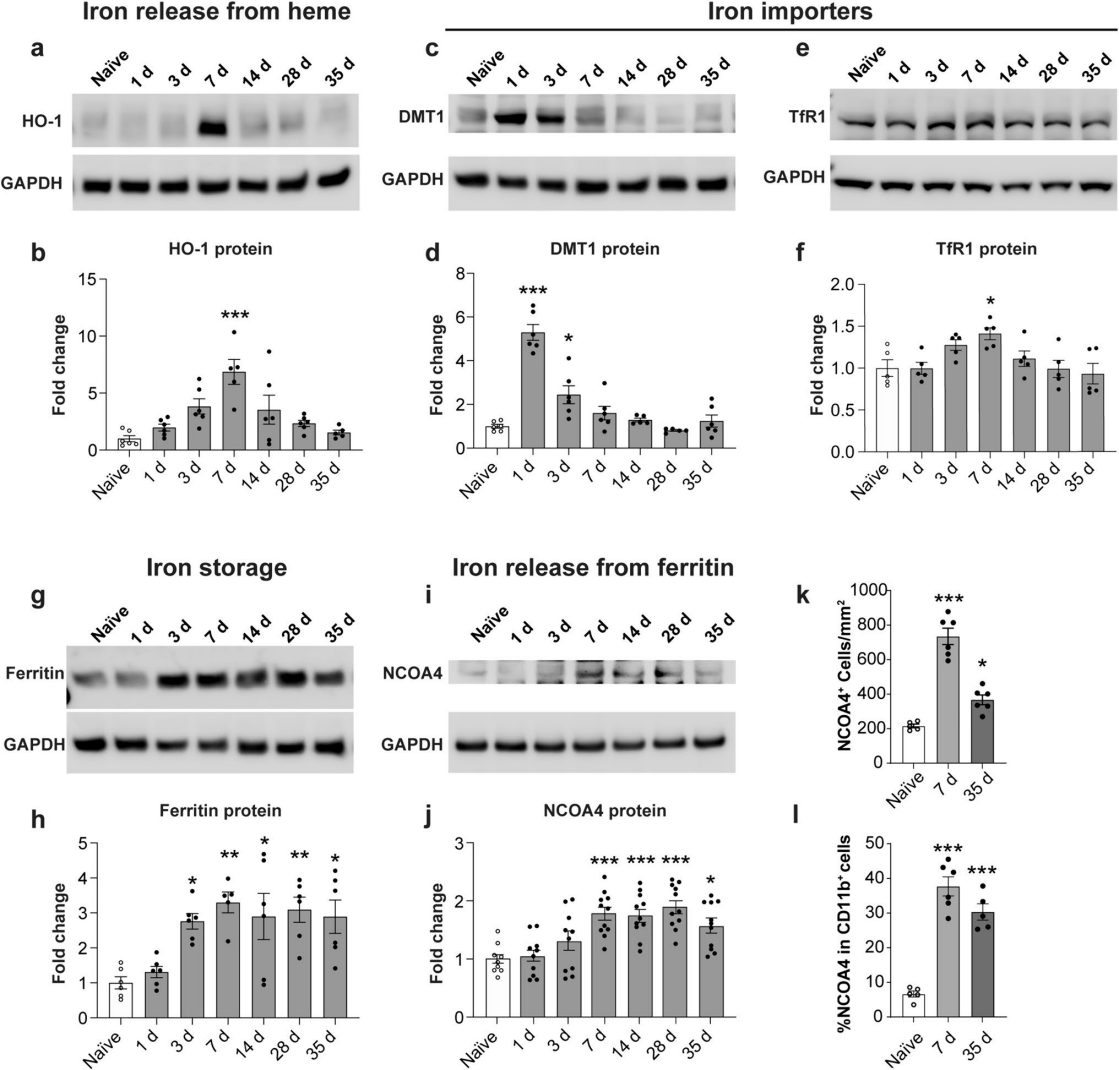
Changes in protein expression of molecules involved in increasing intracellular iron, and iron storage and release from ferritin in mouse SCI tissue. **a**, **b** Western blot data showing changes in expression of HO-1; (Naive vs 7 days: *p* < 0.0001; F(6, 33) = 7.3, *p* < 0.0001, n = 5–6 mice per group). **c**, **d** Expression levels of DMT1 (Naive vs 1 day and 3 days: *p* < 0.0001 and 0.011 respectively; F(6, 33) = 32.32, *p* < 0.0001, n = 5–6 mice per group) and TfR1 (**e**, **f**; Naive vs 7 days: *p* = 0.04; F(6, 28) = 3.8, *p* = 0.006, n = 5 mice per group) as compared to uninjured (naïve) mice. **g**, **h** Expression of ferritin is significantly increased over a longer period (Naive vs 3 days: *p* = 0.03; Naive vs 7 days: *p* = 0.003; Naive vs 14 days: *p* = 0.01; Naïve vs 28 days: *p* = 0.006; Naïve vs 35 days: *p* = 0.016; F(6, 34) = 5.8, *p* = 0.0003, n = 5–6 mice per group). **i**, **j** Likewise with increase in expression of NCOA4 (Naive vs 7d days *p* = 0.0005; Naive vs 14 days: *p* = 0.0009; Naïve vs 28 days: *p* < 0.0001; Naïve vs 35 days: *p* = 0.02; F(6, 66) = 8.98, *p* < 0.0001, n = 9–11 mice per group). **k** Quantification of NCOA4 immunostained cells in the dorsal region of the spinal cord (shown in Fig. 2). (Naive vs 7 days: *p* < 0.0001; Naive vs 35 days: *p* = 0.02; F(2, 14) = 59.80, *p* < 0.0001, n = 5–6 mice per group). **l** Quantification shows that NCOA4 expressed in 37.71 ± 2.75% and 30.31 ± 2.35% of CD11b^+^ cells at 7 days and 35 days after SCI, respectively. (Naive vs 7 days and 35 days: *p* < 0.0001; F(2, 13) = 51.52, *p* < 0.0001, n = 5–6 mice per group). One-way ANOVA with post-hoc Tukey multiple comparison test. *p ≤ 0.05; **p ≤ 0.01 and ***p ≤ 0.001 compared to naïve (uninjured) level. All graphs show Mean ± SEM

**Fig. 2:**
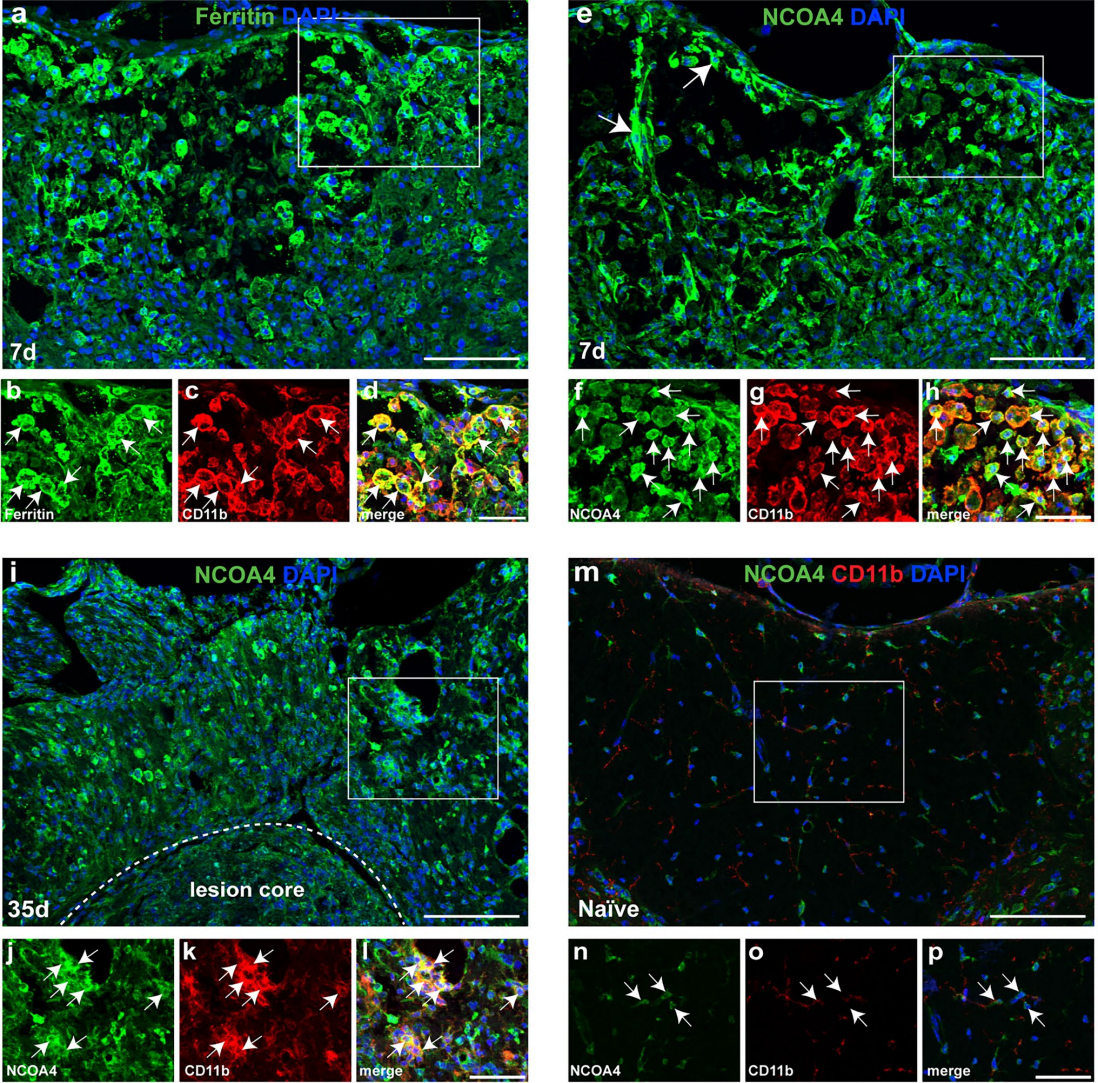
Immunofluorescence labeling for ferritin and NCOA4 after SCI. **a** Shows ferritin labeling (green) and DAPI labeling of nuclei (blue) in a cross-section through the dorsal column of the spinal cord near the lesion epicenter, 7 days after SCI. Double immunofluorescence labeling of the area outlined in the white square in panel **a** is shown in **b** (ferritin), **c** (CD11b) and **d** merged stained images. Note that ferritin is expressed in CD11b + macrophages (arrows in **b**–**d**). **e** Shows NCOA4 labeling (green) and DAPI labeling of nuclei (blue) of an area similar to that in **a** at 7 days post-SCI. Double immunofluorescence labeling of the area outlined in the white square in panel e is shown in **f** (NCOA4), **g** (CD11b) and **h** merged images. Note NCOA4 is expressed in CD11b+ macrophages (arrows in f–h). Panel **i** shows NCOA4 and DAPI staining of cross-section of the spinal cord 35 days post-SCI. Cross-section near the lesion epicenter. Note the lesion demarcated by dashed white line. Split channels of the area outlined in the white square in panel **i** are shown in **j** (NCOA4), **k** (CD11b) and **l** (merged) images. Note CD11b+ macrophages continue to express NCOA4 (arrows) at 35 days post-SCI. The uninjured (naïve) spinal cord shows very weak NCOA4 labeling (**m**). Also shown are the split channels of NCOA4 (**n**), CD11b (**o**) and merged (**p**) labeling. Scale bars = 100 µm; inset = 50 µm

Phagocytosis of RBCs at sites of hemorrhage in the injured spinal cord is thought to be the main contributor to increased intracellular iron in macrophages [7, 8]. The iron contained in heme in hemoglobin is released by the actions of heme oxygenase-1 (HO-1). Increase in immunofluorescence labeling of HO-1 after SCI has been reported in SCI [36]. We show here by Western blots the gradual increase in HO-1 expression that is significantly elevated ~ 6-fold at day 7 post-SCI (Fig. 1a, b) when RBC phagocytosis is at its peak [42]. We also found an increase in immunofluorescence labeling of HO-1 in CD11b+ macrophages after SCI (Supplemental Fig. 1). We also assessed the expression of key molecules involved in iron influx. These include divalent metal ion transporter 1 (DMT1) which imports non-transferrin bound, free iron into cells, and transferrin receptor 1 (TfR1) which imports transferrin-bound iron into cells via a receptor-mediated uptake. Expression of DMT1, a multipass transmembrane transporter which takes up ferrous iron, is increased ~ 5-fold at 24 h post-SCI and ~ 2.5-fold at 3 days post-SCI (Fig. 1c, d). The expression levels remain slightly above normal for the first week (Fig. 1c, d). DMT1 has been reported to be expressed in astrocytes in the injured spinal cord [42]. Expression of TfR1 also appears to be elevated in the first week, reaching significance at 7 days post-injury (Fig. 1e, f). These data show a remarkable increase in expression of HO-1, in addition to increased iron transport proteins DMT1 and TfR1 that mediates increase in intracellular iron. The excess intracellular iron is then stored in ferritin. By Western blots, we detect a statistically significant increase in ferritin expression of between 2.7 and 3.2-fold from 7 to 35 days after SCI (Fig. 1g, h). We also show here that ferritin is expressed in 60.1 ± 1.04% of (n = 6) CD11b+ macrophages in the injured spinal cord at 7 days (Fig. 2a–d).

### SCI induces increased expression of NCOA4 that is involved in iron release from ferritin

Ferrous iron that enters ferritin is oxidized and stored in its inactive mineralized form within the spherical core of ferritin [1]. The expression of ferritin is regulated at the mRNA level by intracellular iron levels [45]. Therefore, the level of ferritin is a good indicator of intracellular iron. Iron required for normal metabolic needs is released from ferritin via ferritinophagy [31]. This requires binding of NCOA4 that shuttles ferritin to autophagosomes for degradation [35] and the release of redox active ferrous (Fe^2+^) iron. Under pathological conditions, increased expression of NCOA4 leads to increased release of redox active ferrous iron that can trigger oxidative damage and ferroptosis. Our Western blots show significantly increased levels of NCOA4 expression from 7 to 35 days after SCI (Fig. 1i, j) Immunofluorescence labeling confirms marked increase in NCOA4 staining after SCI (Fig. 2e) compared to uninjured spinal cord (Fig. 2m). Quantification of NCOA4^+^ cells in the dorsal region of the spinal cord at the lesion epicenter shows a 3.4-fold increase at 7 days (Fig. 1k). NCOA4 labeling is also seen in the ventral white matter (Supplemental Fig. 2). NCOA4 is expressed strongly in 37.71 ± 2.75% of CD11b + rounded macrophages at 7 days post-SCI (Fig. 1l; Fig. 2e and double labeled images in Fig. 2f-h). NCOA4 staining is also seen in some elongated glial cells (likely to be astrocytes) (arrows in Fig. 2e) located near the lesion site. At 35 days after SCI, NCOA4 staining is seen in 30.31 ± 2.35% of CD11b+ macrophages (Fig. 1l) in areas surrounding the lesion core (Fig. 2i–l). No staining was observed in the absence of primary antibody (Supplemental Fig. 3). Compared to the NCOA4 staining after SCI, the normal physiological level of NCOA4 staining is much lower (Fig. 2m–p). This low level is likely required to mobilize iron from ferritin for normal physiological needs. The increased expression of NCOA4 after SCI will lead to release of redox active iron from ferritin. We therefore assessed whether such changes in NCOA4 are associated with increases in redox active Fe^2+^ iron.

**Fig. 3:**
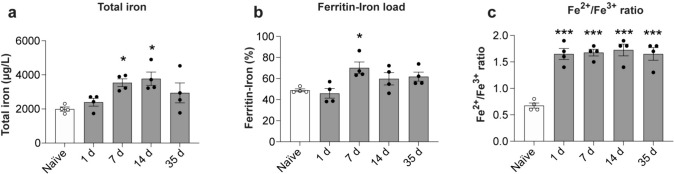
Changes in total iron, iron loading in ferritin and Fe2+/Fe3+ ratio assessed by CE-ICP-DRC-MS. This analysis shows **a** changes in total iron (Naive vs 7 days and 14 days: *p* = 0.04 and 0.018 respectively; *F*_(4, 15)_ = 4.6, *p* = 0.012, *n* = 4 mice per group), and **b** amount of iron loaded into ferritin. (Naive vs 7 days: *p* = 0.03; *F*_(4, 15)_ = 4.66, *p* = 0.012;* n* = 4 mice per group). **c** Shows the rapid and sustained increase in the ratio of Fe^2+^/Fe^3+^ iron in the injured spinal cord (Naive vs 1 day, 7 days, 14 days and 35 days: *p* < 0.0001, *F*_(4, 15)_ = 23.07, *p* < 0.0001; *n* = 4 mice per group). One-way ANOVA with post-hoc Tukey multiple comparison test. *p ≤ 0.05; **p ≤ 0.01 and ***p ≤ 0.001 compared to naïve (uninjured) level

### Changes in total iron content and Fe^2+^ and Fe^3+^ speciation after SCI in mice

We next assessed changes in the total amount of iron in the injured spinal cord tissue, the amount of iron loaded in ferritin, and the ratio of Fe^2+^ to Fe^3+^ iron, using a novel capillary electrophoresis inductively coupled plasma mass spectrometry (CE-ICP-DRC-MS) [38]. Our analysis shows that the total amount of iron in the injured spinal cord increases significantly by ~ 2-fold at 7 and 14-days post-injury, as compared to the uninjured (naïve) spinal cord (Fig. 3a). Interestingly, total iron levels decrease at 35 days post-SCI (Fig. 3a), which may reflect the release of iron from ferritin by ferritinophagy.

As the amount of iron stored in ferritin can vary up to a high of 4500 atoms, we assessed the ferritin-iron load using CE-ICP-DRC-MS. This analysis showed that in the normal, uninjured spinal cord, ferritin contains ~ 45% of its iron storage capacity (Fig. 3b). The iron stored is significantly increased to ~ 65% capacity by 7 days post-injury and remains at about 60% from 2 to 5 weeks post-SCI (Fig. 3b). This data indicates that ferritin in the injured spinal cord has an increased reserve of iron in the first week after SCI that can contribute more iron to ferroptosis particularly in the subacute phase.

Importantly, for ferroptosis to occur, the ratio of Fe^2+^/Fe^3+^ iron needs to be increased. Indeed, this ratio is rapidly increased by ~ 2.5-fold starting at 1 day post-SCI and remains significantly elevated until 35 days post-injury (Fig. 3c). This indicates that increased level of redox active ferrous iron is present during the entire acute to chronic period after SCI, which can contribute to ferroptosis.

### Changes in lipid metabolism markers after SCI in mice

**Fig. 4:**
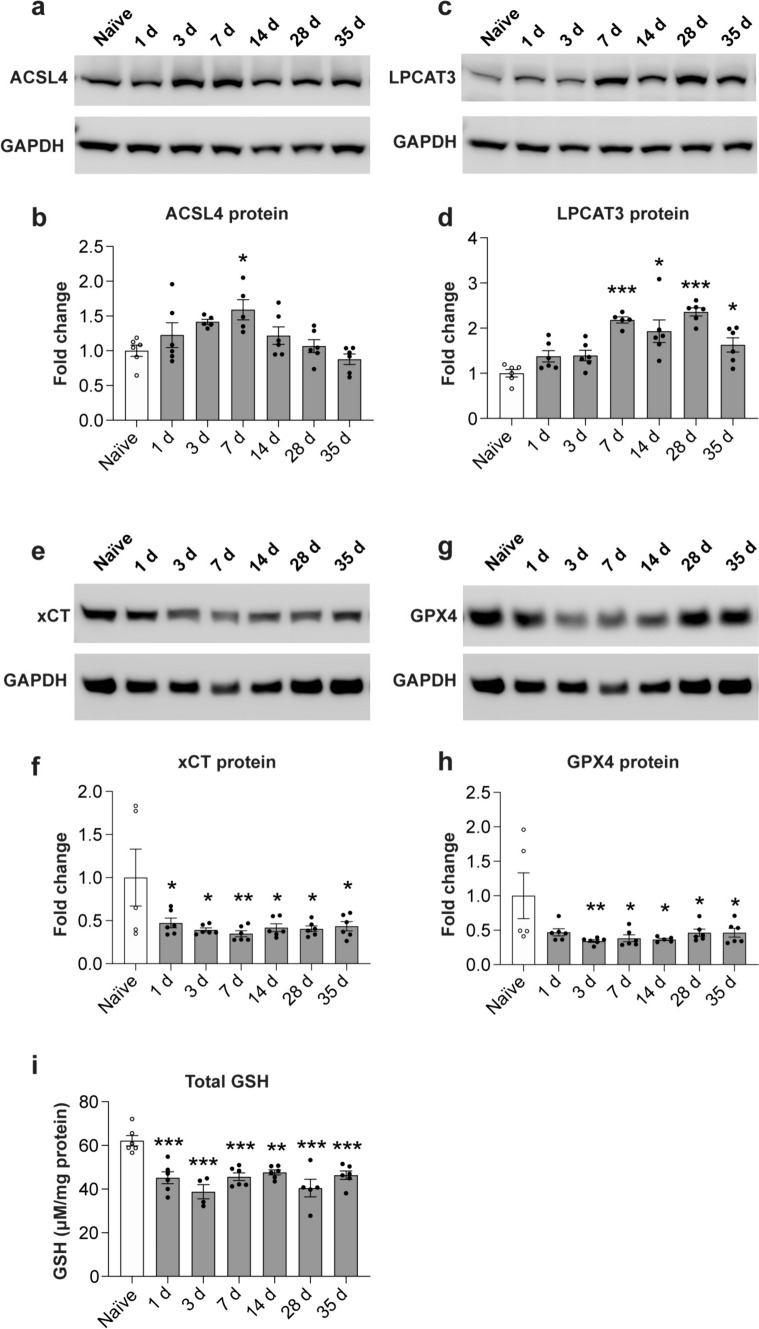
Changes in expression of ferroptosis markers related to lipid repair and GSH metabolism. Western blot analysis of ACSL4 (**a**, **b**) and LPCAT3 (**c**, **d)** show increased expression of ACSL4 at 7 days (**b**), and LPCAT3 at 7–35 days post-SCI (**d**); (**b** ACSL4 Naive vs 7 days: *p* = 0.018;* F*_(6, 33)_ = 4.3, *p* = 0.002,* n* = 5–6 mice per group; **d** Naive vs 7 days: *p* < 0.0001; Naive vs 14 days: *p* = 0.0008; Naïve vs 28 days: *p* < 0.0001; Naïve vs 35 days: *p* = 0.04;* F*_(6, 34)_ = 11.4, *p* < 0.0001,* n* = 5–6 mice per group). Western blotting also shows that system xC (xCT) (**e**, **f**) and GPX4 (**g**, **h**) are significantly reduced in the injured spinal cord from 1–35 d post-SCI as compared to uninjured (naive) spinal cord. (**f** Naive vs 1 day: *p* = 0.04; Naive vs 3 days: *p* = 0.013; Naive vs 7 days: *p* = 0.006; Naive vs 14 days: *p* = 0.019; Naïve vs 28 days: *p* = 0.015; Naïve vs 35 days: *p* = 0.02;* F*_(6, 34)_ = 3.5, *p* = 0.008,* n* = 5–6 mice per group; **h** Naive vs 3 days: *p* = 0.007; Naive vs 7 days: *p* = 0.014; Naive vs 14 days: *p* = 0.016; Naïve vs 28 days: *p* = 0.04; Naïve vs 35 days: *p* = 0.04;* F*_(6, 33)_ = 3.3, *p* = 0.0105,* n* = 5–6 mice per group) (**i**) GSH levels are reduced at all time points examined after SCI as compared to uninjured naïve levels.(Naïve vs 1 day: *p* = 0.0003; Naive vs 3 days: *p* < 0.0001; Naive vs 7 days: *p* = 0.0005; Naive vs 14 days: *p* = 0.002; Naïve vs 28 days: *p* < 0.0001; Naïve vs 35 days: *p* = 0.0009;* F*_(6, 32)_ = 9.3, *p* < 0.0001,* n* = 4–6 mice per group). One-way ANOVA with post-hoc Tukey multiple comparison test. *p ≤ 0.05; **p ≤ 0.01 and ***p ≤ 0.001 compared to naïve (uninjured) level. All graphs show mean ± SEM

Another feature of ferroptosis is the oxidation of membrane phospholipids and the subsequent repair (replacement) of these oxidized fatty acids at the *sn*-2 position that can provide additional new targets for lipid peroxidation and ferroptosis [8]. We therefore assessed changes in expression at the protein level by Western blots, of two enzymes (ACSL4 and LPCAT3) involved in the incorporation of arachidonic acid into membrane phospholipids [29, 44]. Note that these enzymes are known to promote ferroptosis [12, 13]. We find that ACSL4 is significantly increased about 1.5-fold in the first week after SCI (Fig. 4a, b), while LPCAT3 is increased significantly about 2-fold between 7 and 35 days after SCI (Fig. 4c, d). ACSL4 is reported to be expressed ubiquitously in the CNS (in neurons, astrocytes, microglia, oligodendrocytes and endothelial cells) [15]. These results support additional ways in which ferroptosis can be induced after SCI.

### Changes in the glutathione pathway after SCI in mice

A key feature of ferroptosis is that increase in redox active iron needs to be accompanied with an insufficient glutathione antioxidant response. We therefore assessed expression of the cystine-glutamate antiporter (xCT) that couples the export of glutamate to import of cystine into cells [11]. The latter is converted to cysteine and then to glutathione (GSH). Our Western blot analysis revealed a significant rapid reduction in expression of xCT that remained reduced throughout the post-injury period of 35 days (Fig. 4e, f). Quantification of Western blots for GPX4, the enzyme that utilizes GSH to catalyze the reduction of lipid peroxides, showed lower than normal levels significantly from 3 to 35 days (Fig. 4g, h). Note, moreover, that a protective antioxidant response would require an increase in xCT and GPX4, which does not occur. Therefore, lack of increase at all timepoints studied after SCI indicates an insufficient response. Importantly, supporting the previous results, GSH measured using the Glutathione Assay kit from Cayman Chemicals also revealed significant reduction in the levels of GSH from 1 to 35 days post-SCI (Fig. 4i). These results show that there is an insufficient glutathione antioxidant response after SCI in mice that can contribute to ferroptosis and lipid peroxidation.

### Increased lipid peroxidation in the injured mouse spinal cord

**Fig. 5:**
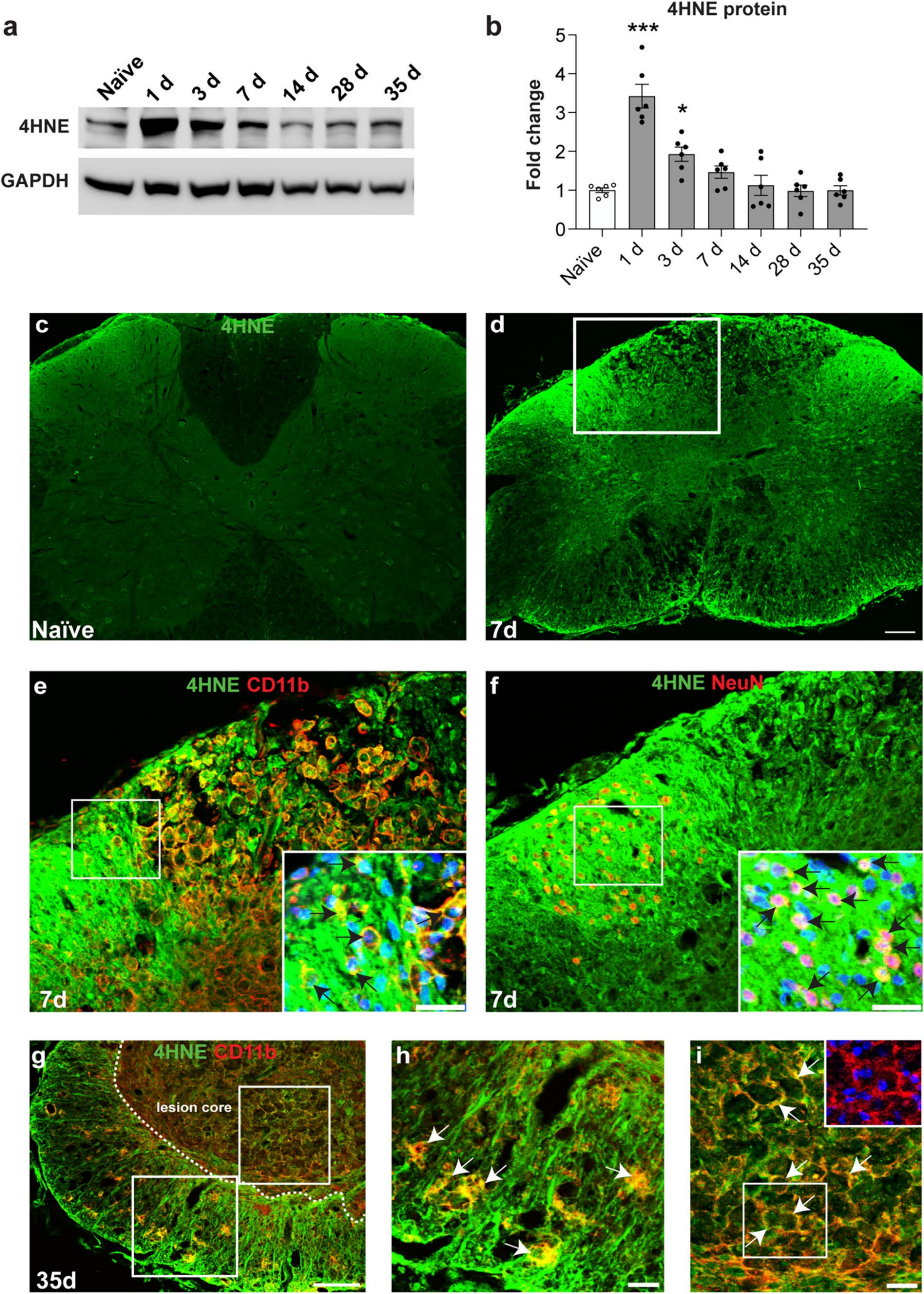
Changes in levels of 4-HNE in the injured mouse spinal cord. **a** Western blot analysis shows that the level of 4-HNE increases after SCI. **b** Significantly increased levels are seen at 1 and 3 days post-SCI (Naive vs 1 day and 3 days: *p* < 0.0001 and 0.02 respectively; *F*_(6, 35)_ = 21.3, *p* < 0.0001,* n* = 6 mice per group, One-way ANOVA with post-hoc Tukey multiple comparison test. *p ≤ 0.05; **p ≤ 0.01 and ***p ≤ 0.001). Graph presents Mean ± SEM. However, immunofluorescence shows there is detectable increase at later time points as compared to uninjured (naïve) controls. Immunofluorescence labeling of 4-HNE in the naïve uninjured spinal cord (**c)** and at 7 days after SCI (**d**). Double immunofluorescence labeling of the dorsal horn region labeled for 4HNE/CD11b (**e**) and 4HNE/NeuN (**f**). Note the double labeled CD11b + macrophages and NeuN + neurons in the insets in e and f, respectively; nuclei in the insets stained with DAPI. **g** Double immunofluorescence labeling of 4HNE/CD11b at 35 days post-SCI. Note the strong 4HNE labeling in CD11b+ macrophages in the white matter and in the lesion core. Regions outlined within the white squares in panel g are shown at higher magnification in panels h (white matter), and i (lesion core). The double labeled cells appear yellow. The large-rounded cells within the lesion core are macrophages as indicated by the single labeling channel for CD11b (red; inset in panel i). The strong 4-HNE labeling in and around the core of the lesion provides evidence of widespread lipid peroxidation that can contribute to oxidative damage even at later time periods (5 weeks) after SCI. Scale bars = 100 µm; inset = 25 µm

We next assessed if the increase in redox active iron and reduction of the antioxidant GSH is associated with increased lipid peroxidation after SCI. Lipid peroxidation of membrane phospholipids in cell membranes, organelles, and myelin generate 4-hydroxy-2-nonenal (4HNE), a highly toxic product. Western blot analysis shows an increase in 4-HNE levels in the injured spinal cord as early as 1 day after SCI and levels remain high during the first week, as compared to uninjured (naïve) mice (Fig. 5a, b). Immunofluorescence analysis additionally shows strong 4HNE staining 7 days post-SCI as compared to minimal labeling in the uninjured naïve spinal cord (Fig. 5c, d). At 7 days post-SCI, robust 4HNE staining is localized to CD11b+ macrophages in the dorsal column white matter adjacent to the site of maximum injury (Fig. 5e). Strong 4HNE labeling is also detected in the adjacent dorsal grey matter localized to CD11b+ macrophages (Fig. 5e) and to NeuN + neurons (Fig. 5f). In addition, lower levels of 4HNE labeling is also seen throughout the spinal cord particularly noticeable in cells that appear to be astrocytes in the ventral white matter (not shown). Unlike the Western blot results, 4-HNE immunostaining is also detected at 35 days post-SCI in CD11b+  macrophages and other glial cells that appear to be astrocytes in the white matter (Fig. 5g, h). This difference in the results between Western blot and immunofluorescence staining may have to do with how certain epitopes are exposed in the two tissue preparations. A dilution effect could also contribute to lower sensitivity of Western blot analysis. CD11b + macrophages within the lesion core also show lower but distinct 4HNE staining that appear to be associated with the cell membranes of CD11b+ macrophages (Fig. 5g, i). Lipid peroxidation therefore occurs in macrophages and other CNS cells after SCI. The widespread lipid peroxidation that persists at 5 weeks post-SCI indicates prolonged, on-going oxidative damage that extends into the chronic period after SCI.

A significant proportion of CC1+ oligodendrocytes near the lesion stain intensely with 4HNE, as compared to the negligible staining in uninjured mice (Supplemental Fig. 4a–d), suggesting that they are likely to be susceptible to ferroptosis. In contrast, there appears to be little overlap of 4HNE staining with GFAP+ astrocytes (Supplemental Fig. 4e–g). This supports the evidence that astrocytes express iron influx and efflux molecules and are well equipped to recycle iron via their perivascular end-feet [8]

### Effects of ferroptosis inhibitor treatment in 40 and 30 kdyne lesions in mice

#### 40 kdyne contusions

**Fig. 6:**
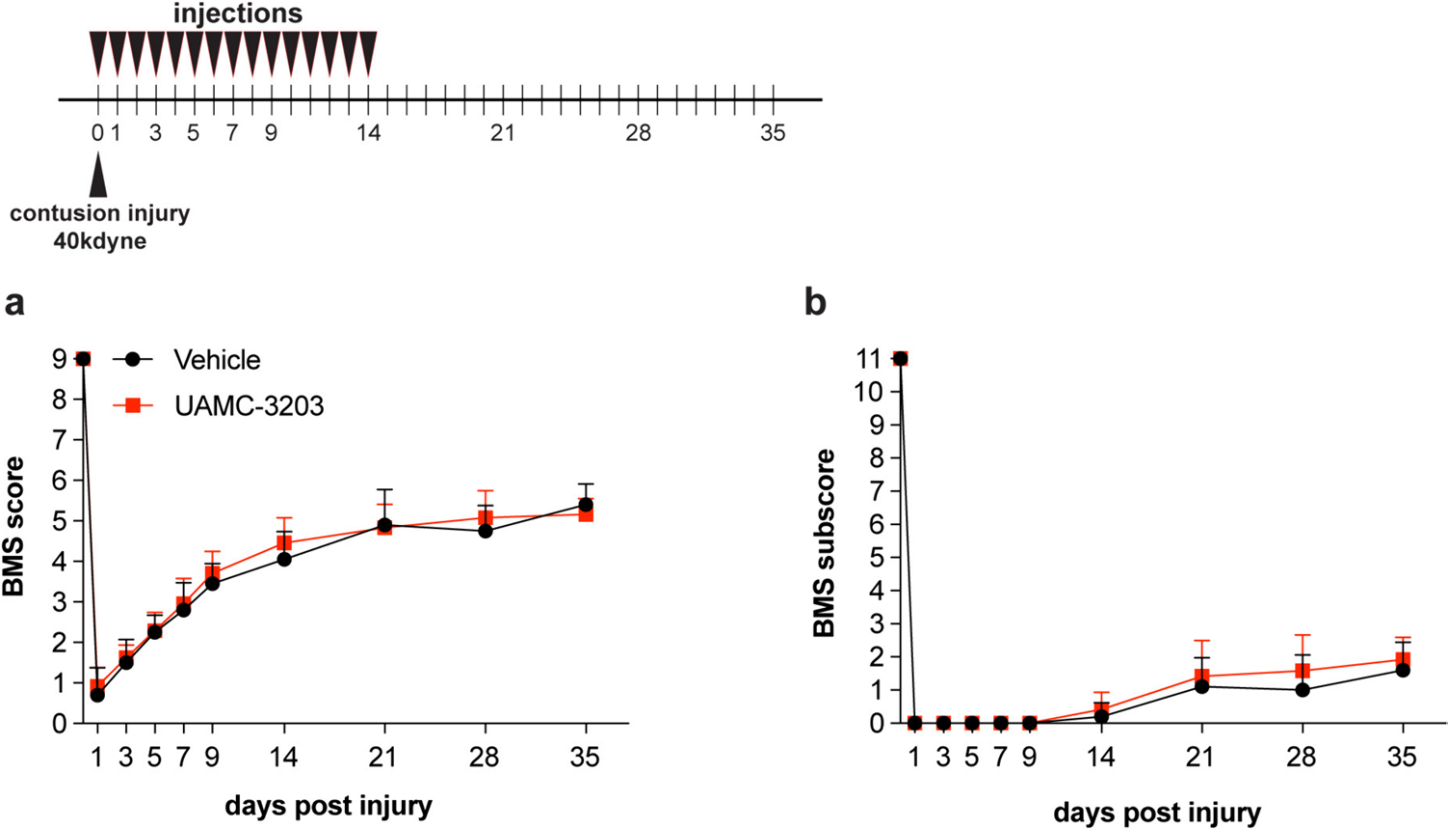
Ferroptosis inhibitor does not improve locomotor recovery after 40 kdyne injury. Locomotor recovery after 40 kdyne injury assessed by BMS analysis **a** shows no improvement after UAMC-3203 inhibitor treatment as compared to control (vehicle) group; (F_(9, 180)_ = 1.22, *p* = 0.28, n = 12 (UAMC-3203) and n = 10 (vehicle)). **b** BMS sub-scores which evaluate finer aspects of locomotor control, also do not show improvement; (F_(9, 180) _= 0.89, *p* = 0.53, n = 12 (UAMC-3203) and n = 10 (vehicle)). Treatment with UAMC-3203 was administered daily for the first 14 days as indicated at the top of panel **a**. All graphs show mean ± SD

We first assessed the effects of ferroptosis inhibitor (UAMC-3203) treatment in mice with 40 kdyne force SCI lesions. Treatments were administered daily for the first 14 days. Inhibitor treatment of 40 kdyne lesions failed to show improvement in locomotor recovery (Fig. 6a). No differences were detected in the BMS scores or subscores, the latter assess finer aspects of locomotor control (Fig. 6b). We have previously observed improved functional recovery with this inhibitor in experimental autoimmune encephalomyelitis (EAE), a mouse model used to study multiple sclerosis, in which iron accumulation and changes in ferroptosis markers occur, as seen in SCI [25]. We therefore decided to test the inhibitor on a slightly lower injury force (i.e., 30 kdyne) lesion of SCI.

#### 30 kdyne contusions

**Fig. 7:**
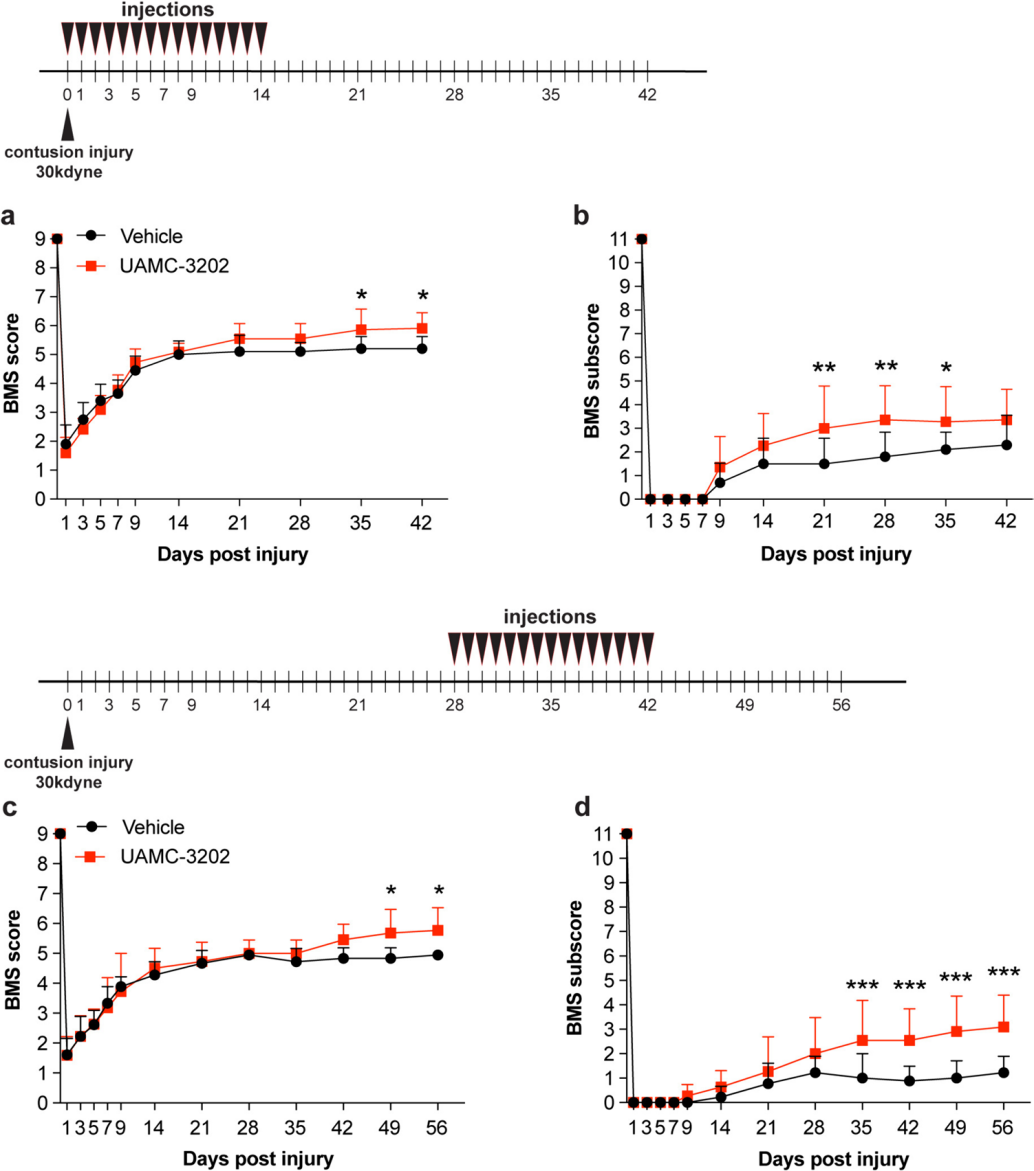
Ferroptosis inhibitor (UAMC-3203) treatment after 30 kdyne injury improves locomotor recovery. Locomotor recovery in the *early treatment group* assessed by BMS analysis **a** shows improvement by 35 and 42 days; (*p*_(35d)_ = 0.02; *p*_(42d)_ = 0.0106;* F*_(10, 190)_ = 4.07, *p* < 0.0001,* n* = 11 (UAMC-3203) and *n* = 10 (vehicle)). **b** BMS sub-scores, which evaluate finer aspects of locomotor control, show significant improvement starting from 21 days post-SCI; (*p*_(21d)_ = 0.003; *p*_(28d)_ = 0.0018;* p*_(35d)_ = 0.04; *F*_(10, 190)_ = 2.7, *p* = 0.003,* n* = 11 (UAMC-3203) and *n* = 10 (vehicle)). In the early treatment group UAMC-3203 was administered daily for the first 14 days as indicated at the top of panel **a**. In the *delayed treatment group* (**c**, **d**), in which treatment was given daily from 28–42 days post-SCI, BMS analysis shows locomotor recovery by days 49 and 56 (**c**); (*p*_(49d)_ = 0.02; *p*_(56d)_ = 0.02;* F*_(12, 216)_ = 2.8, *p* = 0.0012, *n* = 11 (UAMC-3203) and *n* = 9 (vehicle)). **d** Analysis of BMS sub-scores show significant improvement in UAMC-3203 group starting from day 35 onwards* (p*_(35d)_ = 0.0006; *p*_(42d)_ = 0.0002; *p*_(49d)_ < 0.0001; *p*_(56d)_ < 0.0001; *F*_(12, 216)_ = 5.8, *p* < 0.0001, *n* = 11 (UAMC-3203) and *n* = 9 (vehicle)). Two-way RM-ANOVA; time x group effect with post-hoc Bonferroni multiple comparison test. *p ≤ 0.05; **p ≤ 0.01 and ***p ≤ 0.001. All graphs show mean ± SD

The small reduction in injury force from 40 to 30 kdynes was sufficient to result in improved recovery of locomotor control with inhibitor treatment (Fig. 7). This lower force induced significant locomotor deficits as detailed below. Mice were treated with the ferroptosis inhibitor (UAMC-3203; 15 mg/kg body weight) starting either immediately after injury (0–14 days) (early treatment group), or from 28 to 42 days (delayed treatment group). In the *early treatment group*, locomotor recovery assessed using the Basso Mouse Scale (BMS) showed that the ferroptosis inhibitor treatment improved BMS scores starting from day 21 but reached statistical significance on day 35 onwards (Fig. 7a). At 35 days and 42 days the vehicle treated mice showed frequent plantar stepping with no coordination (score, ~ 5). In contrast, mice treated with UAMC-3203 displayed frequent or consistent plantar stepping, with coordination, and parallel paw positioning on contact and lift off (score, ~ 6). BMS sub-scores that evaluate finer aspects of locomotor control showed improvement starting at day 21 (Fig. 7b). Interestingly, the *delayed treatment group* also showed similar improvements in BMS scores starting at day 42 and reached statistical significance on days 49 and 56 (Fig. 7c). BMS sub-scores were improved significantly in the delayed treatment group starting from 35 to 56 days (Fig. 7d). UAMC-3203 treatment at a lower dose of 10 mg/kg body weight had no effect (data not shown) indicating a dose-dependent effect. These data suggest that ferroptosis contributes to functional loss over a wide period from the acute to the chronic period after mild SCI.

#### Comparison of expression of key ferroptosis markers in 30 and 40 kdyne force injuries

**Fig. 8:**
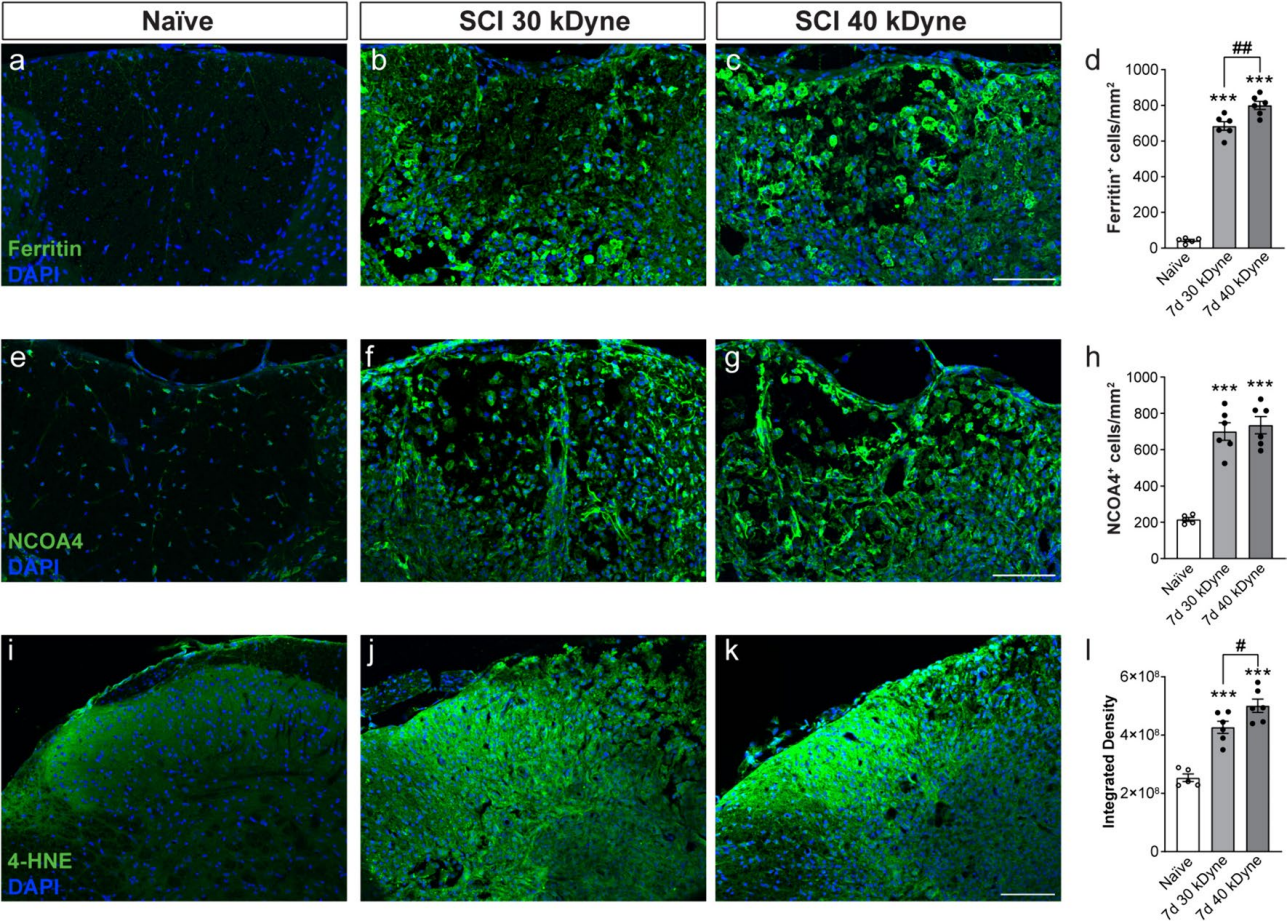
Comparison of the expression of three key markers of ferroptosis after 30 and 40 kdyne force injuries. Expression of ferritin (**a**–**c**), NCOA4 (**e**–**g**), and 4-HNE (**i**–**k**) are increased in the injured spinal cord after both 30 kdyne and 40 kdyne injuries at 7 days after SCI. Quantification shows statistically significant increases after both injuries compared to uninjured controls: **d** Naive vs 7 days 30 kdyne and 40 kdyne: *p* < 0.0001; 7 days 30 kdyne vs 40 kdyne: *p* = 0.003; F_(2, 14)_ = 357.4, *p* < 0.0001, n = 5–6 mice per group). **h** Naive vs 7 days 30 kdyne and 40 kdyne: *p* < 0.0001; 7 days 30 kdyne vs 40 kdyne: *p* = ns; F_(2, 14)_ = 43.8, *p* < 0.0001, n = 5–6 mice per group). **l** Naive vs 7 days 30 kdyne and 40 kdyne: *p* < 0.0001; 7 days 30 kdyne vs 40 kdyne: *p* = 0.04; F_(2, 14)_ = 37.32, *p* < 0.0001, n = 5–6 mice per group). One way ANOVA with post hoc Tukey multiple comparison test. *** *p* ≤ 0.001 compared to naïve (uninjured) level. ^#^*p* ≤ 0.05; ^##^*p* ≤ 0.01 comparing the two force injuries. All graphs show mean ± SEM. Scale bar = 100 μm

Furthermore, we confirmed that key markers of ferroptosis (ferritin, NCOA4 and 4HNE) are also markedly increased in the 30 kdyne force injury, as compared to uninjured spinal cord (Fig. 8). Interestingly, when comparing 30 and 40 kdyne lesions, ferritin and 4HNE showed a small but significant difference, but no difference was detected between 30 and 40 kdyne injuries in expression levels of NCOA4 (Fig. 8), which is considered to be important for triggering ferroptosis.

### Ferroptosis inhibitor treatment reduces secondary tissue damage

The effect of UAMC-3203 treatment in the prevention of secondary tissue damage was assessed in both the early and delayed treatment groups. Several secondary injury parameters were analyzed, including lesion size (tissue loss), myelin sparing, and serotonergic (5-HT) innervation of the ventral horn. Analysis of secondary damage was done on the same cohort of SCI mice (30 kdyne) used for the locomotor analysis.

**Fig. 9:**
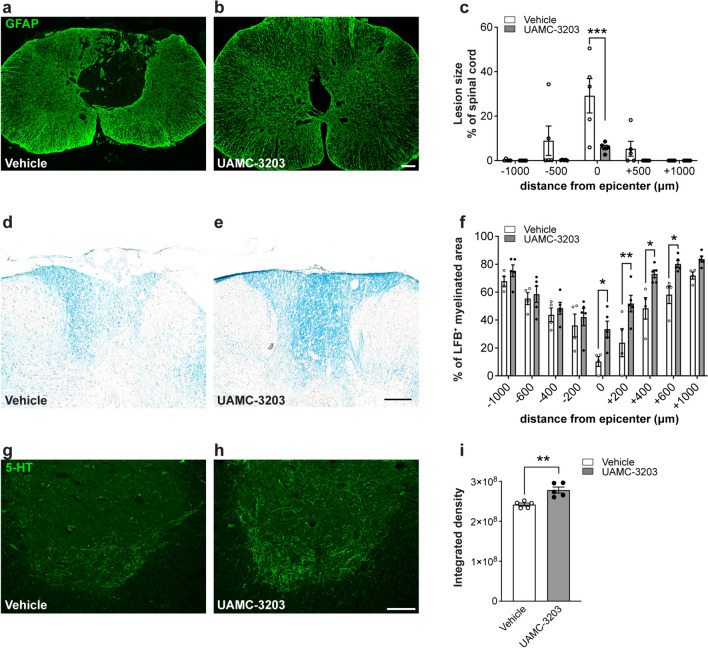
Delayed treatment with ferroptosis inhibitor (UAMC-3203) reduces secondary damage after SCI. All analyses were done on tissue samples from animals used for BMS analysis (30 kdyne) and were obtained on day 56. **a**–**c** Lesion size determined by estimating the area of loss of GFAP staining. Note the larger lesion in the vehicle treated group (**a**) as compared to the UAMC-3203 treated mice (**b**). **c** Quantification shows that the lesion is significantly smaller at the lesion epicenter in the UAMC-3203 treated group as compared to the vehicle treated group. (*p*_(epicenter)_ = 0.0001;* F*_(1, 8)_ = 4.9, *p* = 0.05, *n* = 5 mice per group, Two-way RM-ANOVA; group effect with post-hoc Bonferroni multiple comparison test. *p ≤ 0.05; **p ≤ 0.01 and ***p ≤ 0.001). **d**, **e** Micrographs of LFB staining at the lesion epicenter in vehicle treated SCI mice (**d**) and UAMC-3203 treated SCI mice (**e**). **f** Quantification of LFB staining shows greater myelin at the lesion from epicenter to 600 µm caudally in UAMC-3203 treated group compared to the vehicle group. (*p*_(epicenter)_ = 0.02, *p*_(+200)_ = 0.003, *p*_(+400)_ = 0.015, *p*_(+600)_ = 0.04;* F*_(1, 7)_ = 5.8, *p* = 0.04, *n* = 4–5 mice per group. Two-way RM-ANOVA; group effect with post-hoc Bonferroni multiple comparison test. *p ≤ 0.05; **p ≤ 0.01 and ***p ≤ 0.001). **g**, **h** 5-HT immunoreactivity in the ventral horn region 1 mm caudal to the lesion epicenter shows increased sprouting in UAMC-3203 treated mice (**h**) compared to vehicle treated mice (**g**). **i** Quantification shows significant increase in 5-HT labeling in the UAMC-3203 group compared to vehicle group (vehicle vs UAMC-3203: *p* = 0.007; *n* = 5 mice per group, Two-tailed Mann Whitney U-test; **p ≤ 0.01. All graphs show Mean ± SEM. Scale bars = 100 µm

Lesion size was assessed by staining serial cryostat tissue sections with anti-GFAP antibody. The area of loss of GFAP staining was estimated using the ImageJ software. The delayed treatment group showed that the ferroptosis inhibitor treatment reduced CNS tissue loss at the lesion epicenter by ~ 5-fold) (Fig. 9a–c). Myelin sparing was assessed by staining tissue sections with Luxol fast blue (LFB). Ferroptosis inhibitor treatment significantly increased myelin sparing after delayed treatment at the epicenter by ~ 3-fold and for distances of up to 600 µm caudally (Fig. 9d–f).

We also assessed the effect of the inhibitor treatment on 5-HT innervation of the ventral horn 1 mm caudal to the lesion epicenter. This descending serotonergic innervation from the brainstem is known to play an important role in locomotor control [43]. Mice with SCI in the delayed UAMC-3203 treatment group showed significantly greater serotonergic innervation of the ventral horn 1 mm caudal to the lesion as compared to controls (Fig. 9g–i). This increase in serotonergic innervation caudal to the lesion could be due to sparing of descending 5-HT fibers passing through the lesion epicenter (which show smaller lesion size after inhibitor treatment) or to axonal sprouting.

Furthermore, double labeling for neurofilament and 4HNE, a key marker of ferroptotic damage, in the delayed treatment group shows that many neurofilament + axons are labeled with 4HNE in vehicle treated mice, compared to a 2-fold reduction in the inhibitor treated group (Supplemental Fig. 5). These results suggest that axonal damage may also be reduced by ferroptosis inhibitor treatment.

Similar improvements were also detected in myelin sparing and 5-HT innervation after early (acute) treatment with UAMC-3203 (Supplemental Fig. 6a–f). Collectively these data indicate that ferroptosis contributes to various aspects of secondary damage after SCI.

### Changes in expression of ferroptosis markers in CSF and serum in human SCI

**Fig. 10:**
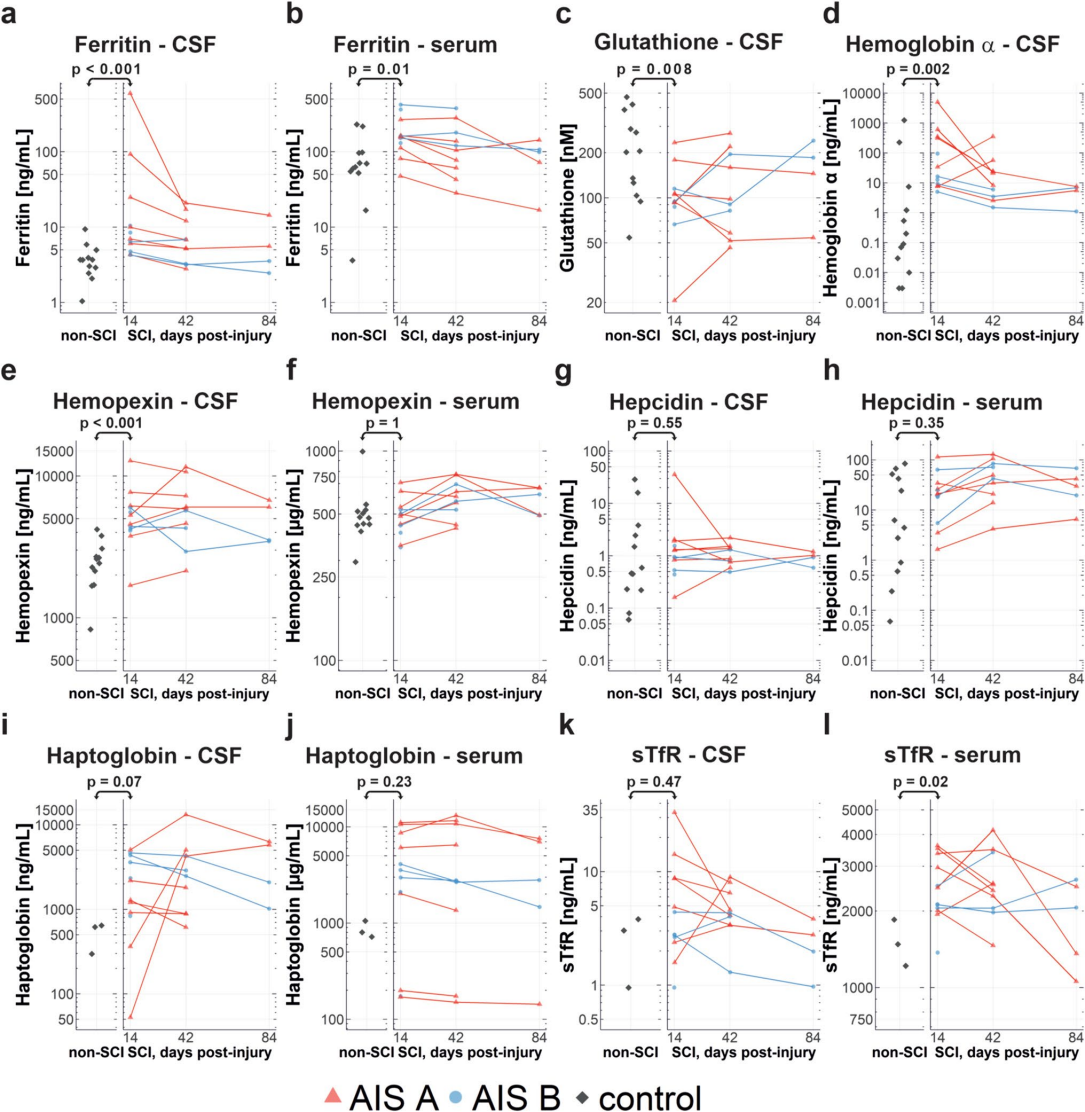
Iron metabolism and ferroptosis and markers in human CSF and serum of SCI and control patients. Dot plots of raw data of ferroptosis markers in control and spinal cord injury groups. Dot plot longitudinal data from spinal cord injury group patients is connected by lines for a better overview. Data is provided for **a** ferritin in CSF, **b** ferritin in serum, **c** glutathione in CSF, **d** hemoglobin α in CSF, **e** hemopexin CSF, **f** hemopexin in serum, **g** hepcidin in CSF, **h** hepcidin in serum, **i** haptoglobin in CSF, **j** haptoglobin in serum, **k** sTfR in CSF, **i** sTfR in serum. Indicated p-values are derived from Mann–Whitney-U-Test results of a comparison of control and 14-days SCI group data. Control group data for haptoglobin and sTfR is available for three patients only, preventing reasonable statistical testing, therefore p-values for these groups should be taken with caution. *AIS* ASIA impairment scale, *CSF* cerebrospinal fluid, *SCI* spinal cord injury, *sTfR* soluble Transferrin Receptor

We carried out enzymatic and immunoassays to quantify the amount of ferritin, hemoglobin α, haptoglobin, hemopexin, soluble transferrin receptor (sTfR) and GSH in the CSF and serum of SCI patients on samples collected from 14 to 84 days after injury. Samples collected from SCI patients with the American Spinal Cord Injury Impairment Scale (AIS) grade A and B were compared to a non-SCI control group (see Table 1 for details). In addition, we calculated Generalized Estimating Equation (GEE) models for each parameter determined in CSF within the SCI group to analyse associations with injury severity (AIS) and time (Table 2). In comparison to control samples, ferritin was significantly higher in SCI patients at 14 days post-injury in CSF (*p* < 0.001, Fig. 10a) and serum (*p* = 0.01, Fig. 10b) compared to controls. The boxplot representation of the comparison between controls and the 14-day timepoint for all markers is shown in Supplemental Fig. 7. Ferritin levels in the CSF of SCI patients were markedly higher at day 14, the earliest time point examined. These values decreased by 42 days and remained at a low level until 84d. GEE models also reveal a clear negative association of CSF-ferritin with time (estimate − 0.779, 95% CI − 1.3891/− 0.168, *p* = 0.012) and some positive association with injury severity (AIS) although lacking statistical significance (estimate 0.955, 95% CI − 0.0086/1.918, *p* = 0.052) (Table 2). A similar time course was also detected with serum samples, but the differences between AIS A and AIS B cases seemed to be inverted with a lower serum ferritin level associated with AIS A (Fig. 10b).

**Table 2:** Association of injury severity and time course on the CSF parameters of spinal cord injured patients

	Estimate	95% CI lower	95% CI upper	p-value
*Ferritin – CSF*				
AIS A (AIS B as reference)	0.955	-0.0086	1.918	0.052
Time (14 d as reference)	-0.779	-1.3891	-0.168	0.012
*Glutathione—CSF*				
AIS A (AIS B as reference)	0.0188	-0.497	0.534	0.940
Time (14 d as reference)	0.1153	-0.204	0.434	0.480
*Hemoglobin—CSF*				
AIS A (AIS B as reference)	2.107	0.903	3.311	< 0.001
Time (14 d as reference)	-1.667	-3.029	-0.305	0.016
*Hemopexin—CSF*				
AIS A (AIS B as reference)	0.1865	-0.233	0.606	0.380
Time (14 d as reference)	0.0748	-0.153	0.302	0.520
*Hepcidin—CSF*				
AIS A (AIS B as reference)	0.574	-0.243	1.391	0.170
Time (14 d as reference)	-0.235	-0.915	0.446	0.500
*Haptoglobin—CSF*				
AIS A (AIS B as reference)	-0.839	-1.539	-0.138	0.019
Time (14 d as reference)	0.689	-0.219	1.596	0.137
*sTfR—CSF*				
AIS A (AIS B as reference)	0.856	0.252	1.461	0.006
Time (14 d as reference)	-0.125	-0.689	0.439	0.663

Generalized Estimated Equation model results for the CSF parameters within the SCI group as dependent (exploratory) variable. Binary variables AIS (A/B) and time (14/42 days post-injury) were used as independent (explanatory) variables

*AIS* American Spinal Injury Association (ASIA) Impairment Scale, *SCI* spinal cord injury, *CSF* cerebrospinal fluid, *CI* confidence interval, *d* days post-injury

Importantly, glutathione levels in the CSF were significantly lower in SCI patients than in controls (*p* = 0.008, Fig. 10c), indicating a reduced glutathione antioxidant defence. Neither SCI severity, nor the time course were associated with CSF glutathione (Table 2).

Hemoglobin α levels in the CSF of SCI patients were significantly higher than in control patients (*p* = 0.002, Fig. 10d) at 14 days post-injury, dropped subsequently but remained higher than half of the controls examined (Fig. 10d). Besides clear changes over time (estimate − 1.667, 95% CI − 3.029/− 0.305, *p* = 0.016), GEE models also show a positive association of hemoglobin α levels with more severe SCI (estimate: 2.107, 95% CI 0.903/3.311, *p* < 0.001) (Table 2).

CSF levels of hemopexin, which binds and transports free heme, a breakdown product of hemoglobin, were higher in SCI patients compared to control patients (*p* < 0.001, Fig. 10e), but no associations with time and severity of injury were observed (Table 2). Serum hemopexin levels were similar between SCI and control patients (Fig. 10f).

CSF and serum levels of hepcidin, a small peptide hormone that binds to the iron efflux transporter ferroportin and causes its internalization and degradation, thus preventing iron release from cells [39], was not different between SCI and control patients (*p* = 0.55, Fig. 10g; *p* = 0.35, Fig. 10h). There was no association of CSF hepcidin with time and the severity of injury (Table 2). Thus, iron retention and accumulation after SCI is not likely to be mediated by hepcidin.

Haptoglobin, which binds free hemoglobin, was higher in the CSF and serum of most SCI patients compared to control samples but did not reach significance (Fig. 10i, j). GEE model data reveal no clear association of CSF haptoglobin with time but an inverted association with SCI severity (Table 2) (estimate: − 0.839, 95% CI − 1.539/− 0.138, *p* = 0.019).

Soluble transferrin receptor (sTfR) is the shed extracellular portion of TfR1 released upon proteolytic cleavage. sTfR binds to transferrin-ferric iron complexes, so can remove bioavailable iron. It is often used as an indicator of iron deficiency anaemia. Its levels were increased 2 to 3.5-fold in serum samples of SCI patients compared to controls (Fig. 10l) and may reflect anaemia in paraplegic patients. In the CSF, its levels were higher in some AIS A patients compared to controls but not in AIS B patients (Fig. 10k). The GEE model results indicate a clear association of sTfR with injury severity (estimate: 0.856, 95% CI 0.252/1.461, *p* = 0.006) but not with time (Table 2).

## Discussion

An important feature of SCI is secondary damage that occurs in the days, weeks and months following injury. A number of factors are thought to contribute to varying extents to secondary damage, including various aspects of the inflammatory response, cell death pathways, oxidative stress and other factors [4–6]. As secondary damage is potentially more amenable to non-invasive therapies, there is much interest in the field to identify factors that contribute to such damage. In our current work we focused our attention on the role of ferroptosis, a form of iron-induced oxidative damage to membrane lipids that leads to tissue damage and cell death [7, 10]. In the lipid-rich environment of the CNS, ferroptosis could therefore play a significant role in secondary damage. Recent studies have assessed the role of ferroptosis in SCI [8]. In the present work, we have extended earlier work on iron in a mouse model of SCI and further analyzed the levels of a range of ferroptosis markers in human CSF and serum after SCI that can serve as useful biomarkers for the treatment and management of SCI in humans.

The key requirements for ferroptosis are (i) availability of excess redox active iron; (ii) reduction in the glutathione antioxidant defense pathway; (iii) the generation of toxic products of lipid peroxidation such as 4-HNE that can damage cell membranes in which it is produced and also spread to adjacent cells to cause widespread damage; and (iv) increased expression of enzymes (ACSL4 and LPCAT3) required for repair of damaged membrane fatty acids that provide new targets for on-going ferroptosis [7]. Changes in the expression of various ferroptosis markers at different times after SCI in mice are summarized in Supplemental Table 1. Additionally, our function-blocking experiments using the next generation ferroptosis inhibitor (UAMC-3203) in mice after SCI, provide credible evidence for a role for ferroptosis in acute and chronic stages of SCI.

TfR1, which is involved in iron uptake into cells, was recently reported to be a ferroptosis marker in a screen of monoclonal antibodies generated by immunizing mice with membrane fractions from lymphoma cells treated with the ferroptosis inducer piperazine erastin [14]. We have recently reported that TfR1 expression increases rapidly at the onset of the chronic form of EAE, a mouse model of multiple sclerosis (MS) [25]. In SCI, on the other hand, we detect only a small increase in TfR1 in the first week after injury in which hemorrhage occurs and phagocytosis of RBCs is the major source of iron [42]. RBCs induce a heavy iron load in macrophages, as each RBC can hold about one billion atoms of iron within hemoglobin [8]. Iron is extracted from heme by HO-1. We now confirm and extend previous reports of HO-1 expression in macrophages after SCI [36] and show that expression of HO-1 peaks at 7 days post-SCI indicating that this is when much of the iron from heme in RBCs is likely to be extracted and stored in ferritin. In addition, iron can also be taken up into cells via DMT1, a divalent metal ion transporter, which we show is increased early after SCI. We have shown previously that DMT1 is expressed in astrocytes [23, 36, 42], which are capable of recycling iron out of the CNS via astrocytic end-feet around blood vessels [8]. Iron released from lysed RBCs in the tissue at the site of hemorrhage can also generate free radicals and induce ferroptosis after SCI. Interestingly, in the CSF of human SCI cases, we detected increased levels of hemoglobin α, and hemopexin (which binds free heme), suggesting that these can be used as useful biomarkers in SCI to detect toxic iron released from hemoglobin that can contribute to ferroptosis.

Excess iron in macrophages is stored safely in ferritin, which our Western blots show is significantly increased from 3 to 35 days after SCI (the maximum time-period examined). Ferritin expression is regulated at the mRNA level via the iron regulatory protein-iron regulatory element (IRP-IRE) system based on intracellular iron levels. The level of ferritin is therefore a very good indicator of cytoplasmic iron levels. Interestingly, we show here that human CSF from SCI AIS A patients show elevated levels of ferritin in the first 42 days post-lesion that could serve as a biomarker for increased iron and severity of injury. Additionally, conventional MRI and newer quantitative MRI protocols can also be used to detect iron deposits and hemorrhage in the injured human spinal cord [16, 47]. A 2-year MRI study of SCI patients also reported progressive neurodegeneration and iron accumulation in supraspinal regions as early as 6 months after SCI and is a predictor of worse neurological outcomes [58]. Our novel CE-ICP-DRC-MS analysis also shows a significant increase in redox active ferrous iron from 1 day to 5 weeks after SCI in mice, a key requirement for ferroptosis.

A reduced antioxidant (GSH) response is another essential requirement for ferroptosis [46, 51]. This includes reduction in xCT, GSH and GPX4 [11, 51]. After SCI in mice, we detected a rapid and sustained reduction of system xCT an antiporter required to transport cystine into cells for the production of GSH; reduction of GSH for the entire 5-week period after SCI; and reduction of the enzyme GPX4 that utilizes GSH to protect membrane phospholipids against iron-catalyzed lipid peroxidation and prevent accumulation of toxic lipid radicals [22, 51]. Previous studies reported reduction of xCT, GPX4 and GSH in the first 2 weeks after SCI in rodent models [26, 30, 55, 56]. We have now extended these findings to show sustained reduction of all three markers for up to 5 weeks post-injury in mice. Moreover, the CNS has relatively low levels of GSH as compared to other organs, making it more susceptible to oxidative damage and ferroptosis [32, 48]. Reduction of GSH and GPX4 levels in CSF and serum has been reported in human SCI [3]. Our findings of lower GSH levels in the CSF of SCI patients from 14–84 days support and extend the earlier reports.

Another important indicator of ferroptosis is the increase in toxic aldehyde end-products of lipid peroxidation that includes 4-HNE. We detected rapid and sustained increase in 4-HNE from 1 day to 5 weeks post-SCI in mice. We have previously reported increased 4-HNE staining 42 days after SCI in mice [42]. Other studies in rats also reported increase in 4-HNE from 1 h to 7 days (longest time point examined) [50, 53], and 14 days after SCI [56]. Increased 4-HNE has been shown to be associated with reduction in CSF levels of GSH and GPX4 in SCI patients [56]. Our current work also shows reduced GSH levels in the CSF for up to 84 days post-SCI in humans. These indicators could be useful CSF biomarkers but will need additional studies to assess and confirm how such changes correlate with severity of the lesion, and time after injury.

Other elements that can sustain on-going ferroptosis in the chronic period, several weeks after SCI, are the lipid repair enzymes ACSL4 and LPCAT3. ACSL4 controls the level of arachidonic acid (AA) in cell membranes by regulating arachidonoyl-CoA synthesis; and deletion of ACSL4 has been shown to prevent ferroptosis [13, 49, 54]. LPCAT3 on the other hand catalyzes the insertion of acylated AA into membrane phospholipids. These two enzymes therefore replace oxidized fatty acid (arachidonic acid) at the sn-2 position in cell membranes and thus provide additional new targets for on-going ferroptosis. We report that expression of ACSL4 and LPCAT3 are both increased in mice between 7 and 35 days after SCI. They can therefore contribute to maintaining continued ferroptosis in the acute and chronic period after SCI.

The human data provide some interesting insights into changes in expression of iron metabolism and ferroptosis markers after SCI. It is important to note, however, that it is difficult to make direct comparisons between mouse and human SCI data. Given the availability and logistics of acquiring human SCI samples, the number of cases is small. Moreover, unlike the targeted mild lower thoracic lesion in young adult, female mice, the human samples are from cases ranging in age from 16 to 75 years, with lesions at C4-C6 levels, and motor complete ASIA A and B impairment scale. Nevertheless, this pilot human data on iron related ferroptosis markers provides valuable information, which needs to be pursued further with more detailed analysis of different severities and types of spinal cord lesions.

The effects of ferroptosis inhibitors (SRS 16–86 and ferrostatin-1) have been assessed in adult rats at early (acute) time points (1 and 7 days after SCI) [55, 57]. Such treatment resulted in improvement in locomotor recovery and secondary damage in acute SCI. In another study, 10 µl of ferrostatin-1 was injected into the injured rat spinal cord (on day 1 and 2 after injury) also reduced white matter damage and resulted in modest improvement in locomotor control [17]. In the current study, we have extended these studies to assess both the acute and chronic effects of a third generation ferroptosis inhibitor (UAMC-3203 HCl) which has better solubility and pharmacokinetics than ferrostatin-1 (the parent compound) [9]. While acute treatment did not improve locomotor function after moderate SCI, both early treatment (0–14 days post-SCI) and delayed treatment (28–42 days post-SCI) improved locomotor recovery after mild SCI (30 kdyne). We recently reported that UAMC-3203 improves motor function and reduces tissue damage in a mouse model of chronic experimental autoimmune encephalomyelitis (EAE) in mice, a model that has relevance for secondary progressive MS [25]. Improvement in more severe SCI may be possible as more potent ferroptosis inhibitors are developed and tested. Moreover, the modest functional improvement in SCI we have observed aligns with the concept currently accepted in the field that multiple factors contribute to secondary damage after SCI and that manipulation of only one target is not sufficient to elicit substantial recovery [20]. Achieving this will require combination therapies, for which the identification of individual targets is essential. Ideally such combination therapies will need to target different molecular pathways that contribute to secondary damage, including ferroptosis. Another important take home message from this work is that ferroptosis continues to occur in the chronic phase after SCI as delayed treatment is effective in reducing neurodegenerative changes.

### Supplementary Information

Below is the link to the electronic supplementary material.

**Table S1.** Summary of the mean, SEM, p-values and other statistical data for changes in expression of various molecules at different times after SCI (PDF 39721 KB)
