# Supplementary material for: Ferroptosis inhibitor improves outcome after early and delayed treatment in mild spinal cord injury
Source: Acta Neuropathol. 2024 Jun 22;147(1):106. doi: 10.1007/s00401-024-02758-2 (PMC11193702; doi:10.1007/s00401-024-02758-2)
Supplement: Supplementary file 1 — Table S1. Summary of the mean, SEM, p-values and other statistical data for changes in expression of various molecules at different times after SCI (PDF 39721 KB) [file 401_2024_2758_MOESM1_ESM.pdf]

Supplemental figures and legends 1 - 7
Supplemental table 1

Ferroptosis inhibitor improves outcome after early and delayed treatment in mild spinal cord injury

Fari Ryan, Christian Blex, The Dung Ngo, Marcel A Kopp, Bernhard Michalke, Vivek Venkataramani, Laura Curran, Jan M. Schwab, Klemens Ruprecht, Carolin Otto, Priya Jhelum, Antje Kroner, and Samuel David

Supplemental Figure 1

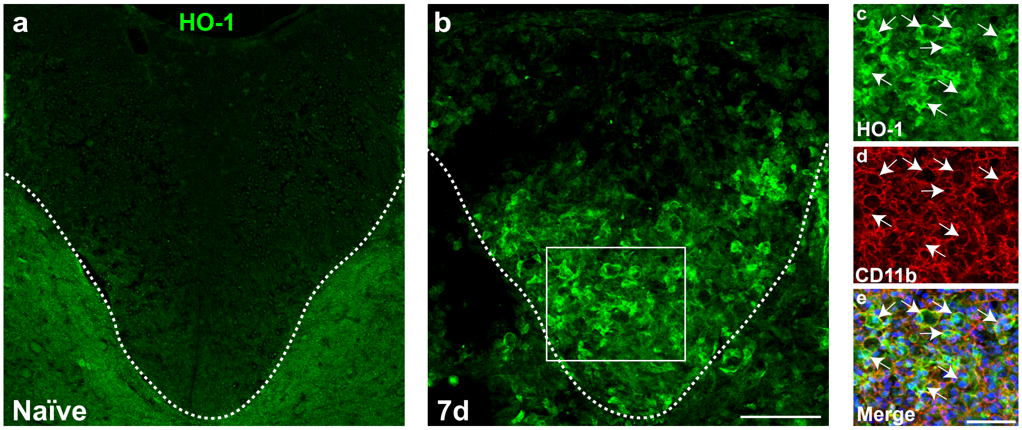

Micrographs showing evidence of HO-1 expression in CD11b$^+$ macrophages after SCI. Immunofluorescence labeling for HO-1 (green) in the dorsal region of the uninjured **(a)** and injured spinal cord 7d after 40kdyne injury **(b)**. The area of the dorsal column white matter is outlined in dashed white lines. The area outlined in the white square in (b) is shown in higher magnification and split color images in panels c, d, e, to show double labeling of HO-1 (green; arrow in panel c) in CD11b$^+$ (red; arrows in panel d macrophages. The merged image showing double labeled cells is shown in panel e (arrows). Scale bars in b = 100 μm, and e = 25 μm

Supplemental Figure 2

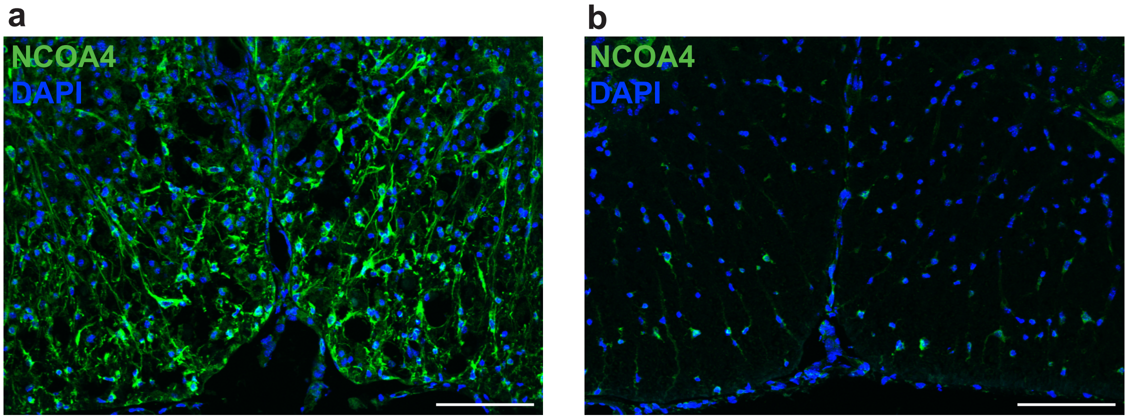

NCOA4 staining of ventral spinal cord 7d after SCI (40kdyne). Note the abundance of NCOA4 staining (green) in the ventral white matter at 7d after SCI **(a)** compared to uninjured controls **(b)**. Sections stained with DAPI to label nuclei. Scale bar = 100 µm

Supplemental Figure 3

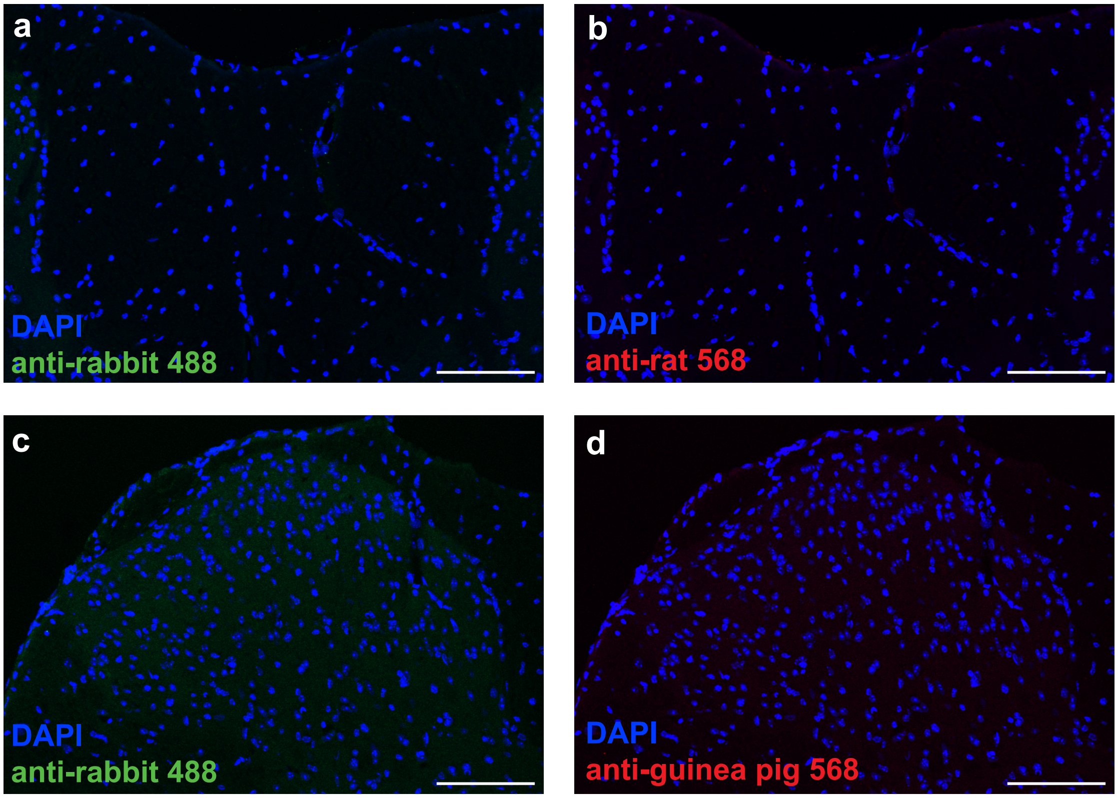

Micrographs of sections stained with no primary antibody.

No staining is observed in the absence of primary antibody. **(a, b)** Show section incubated without primary antibody but double labeled with anti-rabbit 488 (a) and anti-rat 568 (b). **(c, d)** Shows section incubated without primary antibody but double labeled with anti-rabbit 488 (c) and anti-guinea pig 568 (d). Sections are stained with DAPI. Scale bar = 100μm.

Supplemental Figure 4

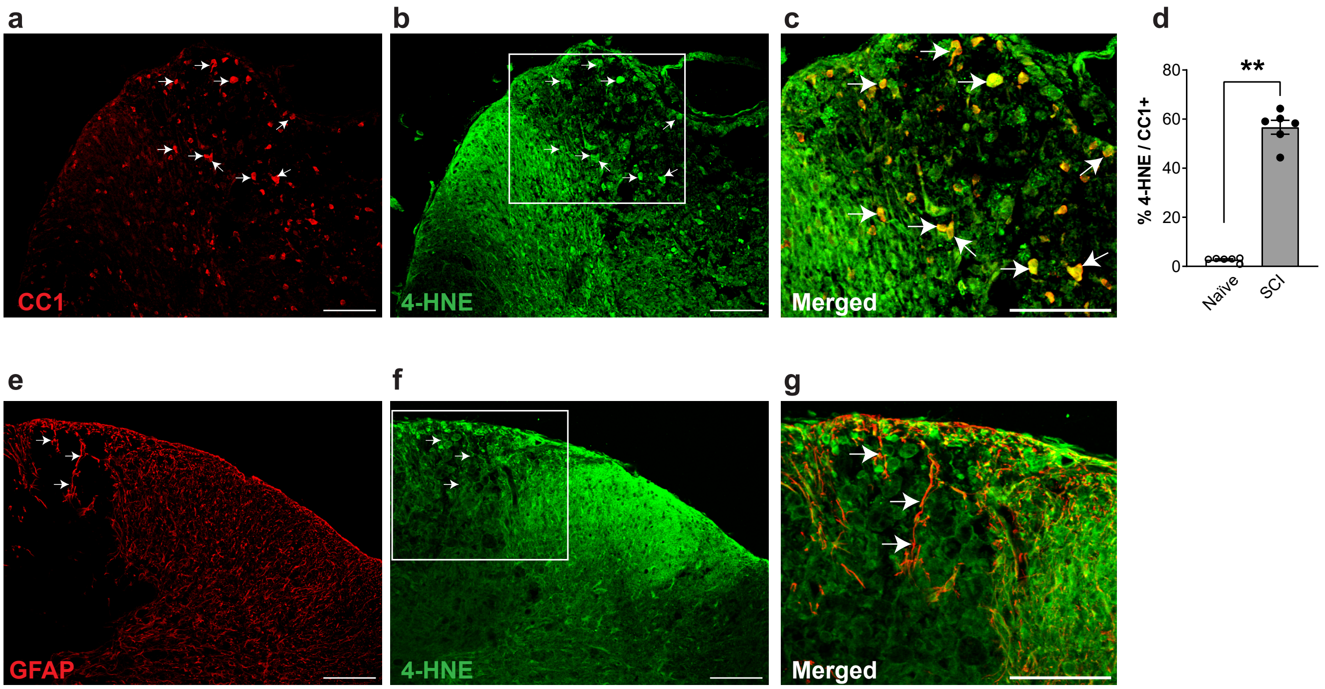

Double immunofluorescence labeling of CC1/4HNE and GFAP/4HNE. (a-c) Micrographs showing CC1 (a) and 4HNE (b) labeling of the dorsal region of the injured spinal cord 7 days after SCI (40kdyne). Note the large number of double labeled cells (yellow in panel c, arrows). **(d)** Quantification shows that 56.6 ± 2.83% of CC1^{+} oligodendrocytes are 4HNE^{+} (Naïve vs SCI: p = 0.002; n = 6 mice per group, Two-tailed Mann Whitney U-test. **p ≤ 0.01). **(e-g)** Micrographs showing GFAP (e) and 4HNE (f) labeling of the dorsal region of the spinal cord 7d after SCI. Note that most of the GFAP^{+} astrocyte profiles are not labeled with 4HNE (g, arrows). Areas outlined in white squares in panels b and e are shown at higher magnification in panels c and f. Scale bar = 100 μm

Supplemental Figure 5

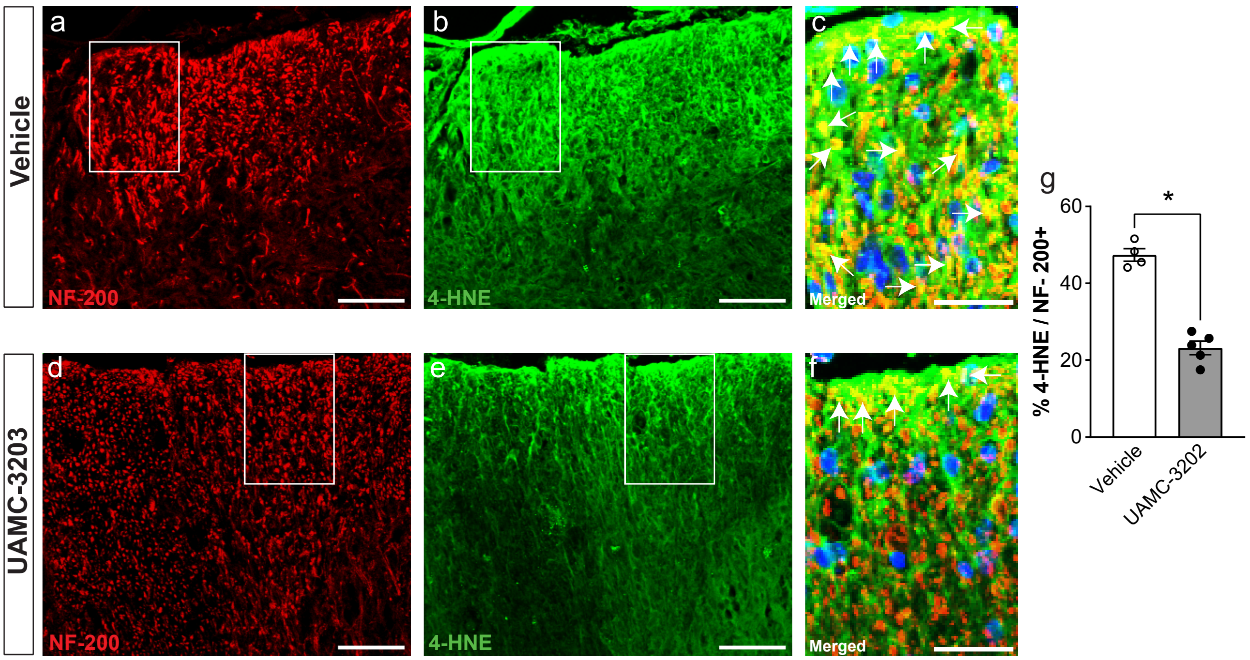

Delayed treatment with UAMC-3203 reduces 4HNE labeling of axons after SCI. Double immunofluorescence labeling of the dorsal region of the spinal cord (30 kdyne), labeled for neurofilament (NF-200) **(a, d)** and 4-HNE **(b, e)** in vehicle **(a-c)** and UAMC-3203 treated **(d-f)** mice. Note that there are more double labeled axons (yellow; arrows) in vehicle treated **(c)** than in the inhibitor treated (**f**; arrows) mice. Note that the areas outlined in the white squares in panels a, b and d, e are shown in higher magnification in panels c and f, respectively. Quantification of double labeled axons **(g)** shows a 2-fold reduction in 4-HNE^{+} axons after ferroptosis inhibitor treatment (Vehicle vs UAMC-3203: $p = 0.016$; $n = 4$-5 mice per group, Two-tailed Mann Whitney U-test. $^{*}p \leq 0.05$). Scale bar = 50 μm; inset = 25 μm.

Supplemental Figure 6

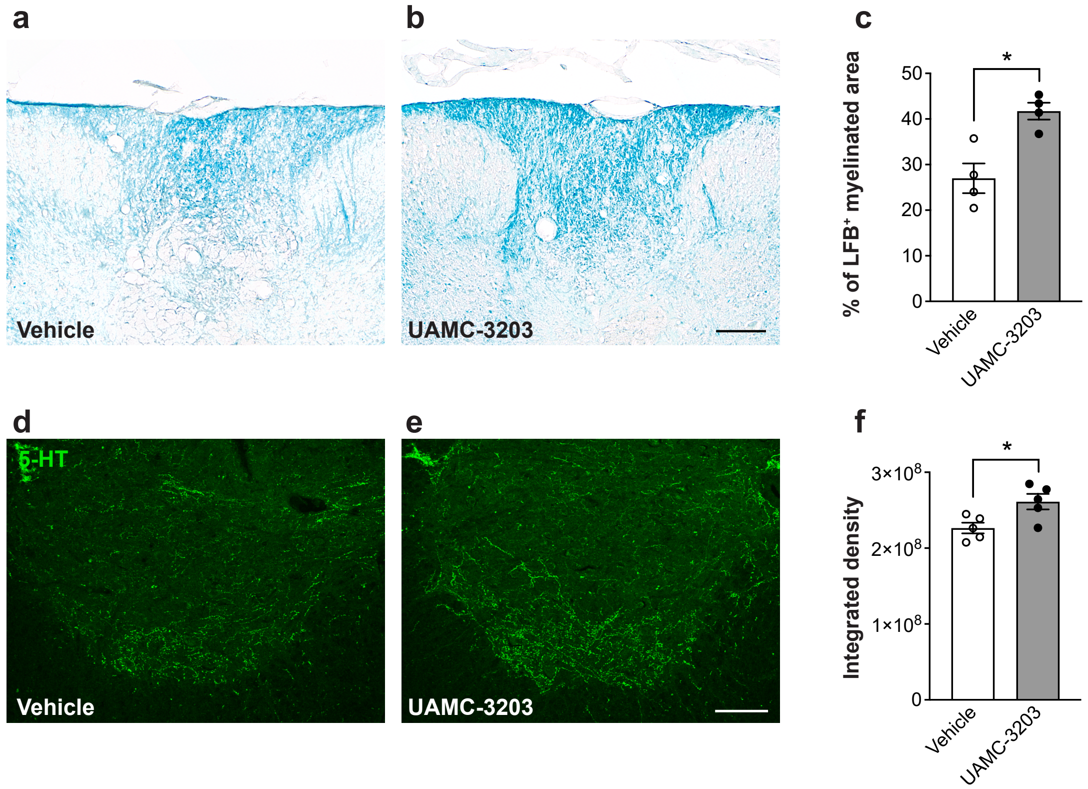

Micrographs showing effects of early (acute) treatment with UAMC-3203 on staining for LFB (myelin) and 5-HT innervation in injured spinal cord (30 kdyne). LFB staining of vehicle treated **(a)** and UAMC-3203 treated **(b)** mice in the acute treatment group. Note the greater sparing of myelin in the UAMC-3203 treated animal. **(c)** Quantification of LFB staining shows significantly greater myelin sparing at the lesion epicenter in UAMC-3203 treated group compared to the vehicle group. (Vehicle vs UAMC-3203: p = 0.02; n = 4 mice per group, Two-tailed Mann Whitney U-test). **(d, e)** Micrographs of 5-HT immunoreactivity in the ventral horn region 1 mm caudal to the lesion epicenter in vehicle treated (d) and UAMC-3203 treated (e) animals in the acute treatment group. Note increased 5-HT innervation in UAMC-3203 treated mice compared to vehicle treated mice. **(f)** Quantification shows significant increase in 5-HT labeling in the UAMC-3203 group compared to vehicle group in the acute treatment group (Vehicle vs UAMC-3203: p = 0.03; n = 5 mice per group, Two-tailed Mann Whitney U-test). $^*p \leq 0.05$. Scale bar = 100 μm.

Supplemental Figure 7

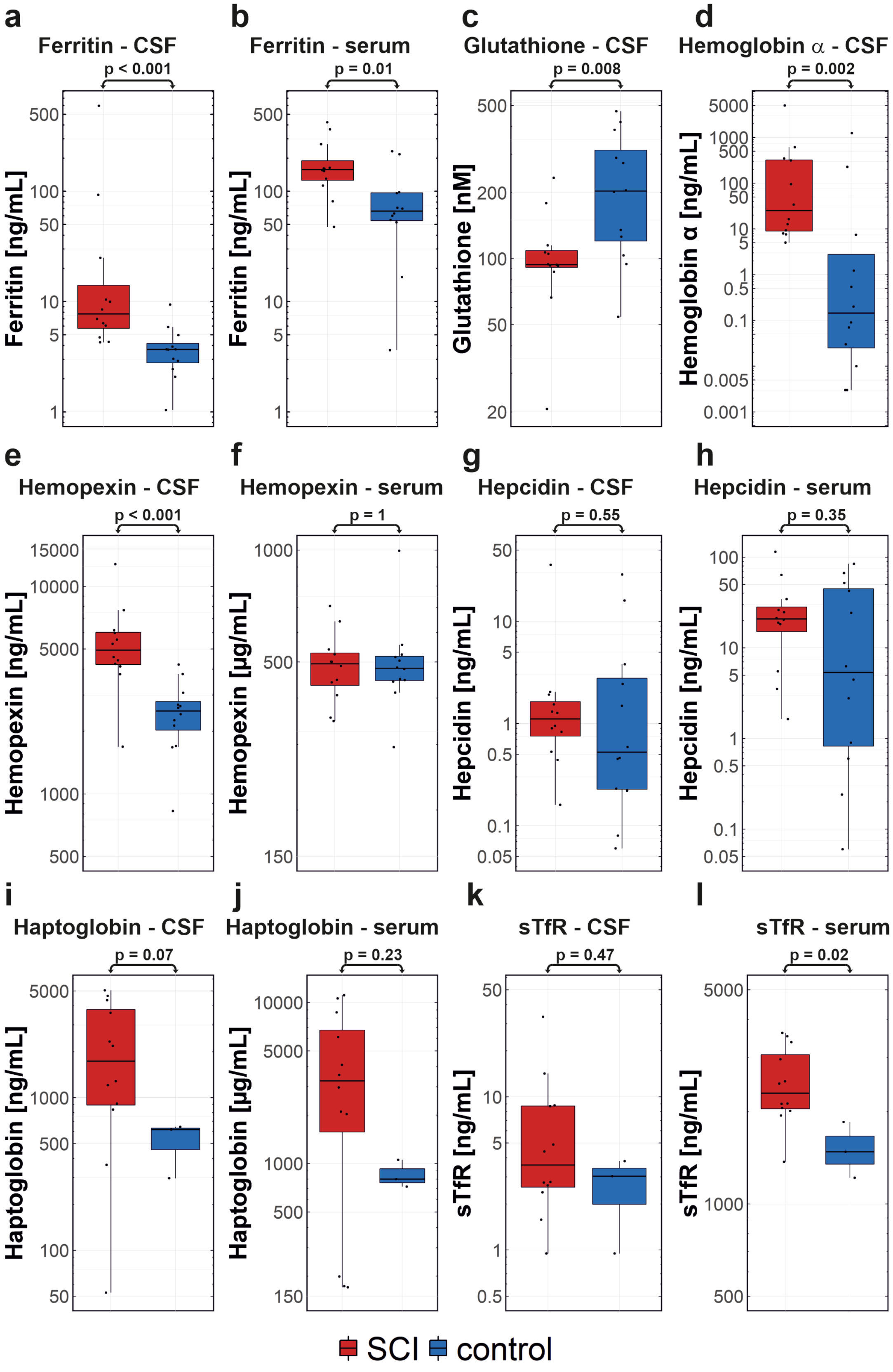

Boxplot representation of statistical analysis of 14-day time point of ferroptosis markers in human CSF and serum. Comparison of iron metabolism and ferroptosis markers in the SCI group at 14 days post-injury and control group using Tukey box plots. Data is provided for **a**: ferritin in CSF, **b**: ferritin in serum, **c**: glutathione in CSF, **d**: hemoglobin α in CSF, **e**: hemopexin CSF, **f**: hemopexin in serum, **g**: hepcidin in CSF, **h**: hepcidin in serum, **i**: haptoglobin in CSF, **j**: haptoglobin in serum, **k**: sTfR in CSF, **l**: sTfR in serum. Indicated p-values are derived from Mann-Whitney-U-Test results. Control group data for haptoglobin and sTfR is available for three patients only, preventing reasonable statistical testing, therefore p-values for these groups should be taken with caution. Abbreviations: AIS = ASIA impairment scale, CSF = cerebrospinal fluid, SCI = spinal cord injury, sTfR: soluble Transferrin Receptor.

Supplemental table 1

	proteins	naive	days post spinal cord injury												ANOVA		
			1 d		3 d		7 d		14 d		28 d		35d		F ratio	P value	Effect size (η^2)
		m (sem)	m (sem)	p	m (sem)	p	m (sem)	p	m (sem)	p	m (sem)	p	m (sem)	p			
Western blot	HO-1	1 (0.27)	1.99 (0.30)	0.95	3.84 (0.66)	0.08	6.87 (1.10)	<0.0001	3.55 (1.27)	0.15	2.34 (0.27)	0.81	1.55 (0.22)	0.99	7.30	<0.0001	0.57
	DMT1	1 (0.09)	5.29 (0.36)	<0.0001	2.44 (0.41)	0.011	1.60 (0.31)	0.71	1.29 (0.09)	0.99	0.81 (0.04)	0.99	1.24 (0.28)	0.99	32.32	<0.0001	0.85
	TFR1	1 (0.10)	1 (0.07)	>0.99	1.27 (0.06)	0.36	1.41 (0.07)	0.04	1.11 (0.09)	0.98	0.99 (0.10)	>0.99	0.93 (0.12)	0.99	3.80	0.006	0.45
	Ferritin	1 (0.17)	1.31 (0.16)	0.99	2.76 (0.22)	0.03	3.30 (0.30)	0.003	2.90 (0.66)	0.016	3.09 (0.36)	0.006	2.89 (0.48)	0.016	5.84	0.0003	0.51
	NCOA4	1 (0.08)	1.06 (0.11)	0.99	1.31 (0.17)	0.60	1.78 (0.11)	0.0005	1.76 (0.11)	0.0009	1.91 (0.11)	<0.0001	1.59 (0.12)	0.02	8.98	<0.0001	0.45
	ACSL4	1 (0.08)	1.23 (0.18)	0.79	1.42 (0.04)	0.19	1.59 (0.15)	0.018	1.22 (0.12)	0.81	1.07 (0.09)	0.99	0.88 (0.07)	0.98	4.34	0.002	0.44
	LPCAT3	1 (0.08)	1.38 (0.13)	0.50	1.40 (0.12)	0.44	2.18 (0.07)	<0.0001	1.93 (0.25)	0.0008	2.36 (0.09)	<0.0001	1.63 (0.16)	0.04	11.48	<0.0001	0.67
	xCT	1 (0.33)	0.47 (0.06)	0.04	0.39 (0.02)	0.013	0.35 (0.03)	0.006	0.42 (0.04)	0.019	0.41 (0.03)	0.015	0.44 (0.05)	0.02	3.51	0.008	0.38
	GPX4	1 (0.33)	0.47 (0.05)	0.051	0.34 (0.02)	0.007	0.38 (0.05)	0.014	0.37 (0.02)	0.016	0.46 (0.05)	0.04	0.46 (0.06)	0.04	3.38	0.011	0.38
	4-HNE	1 (0.06)	3.42 (0.30)	<0.0001	1.93 (0.18)	0.02	1.46 (0.16)	0.61	1.13 (0.26)	0.99	0.98 (0.15)	0.99	1 (0.12)	0.99	21.38	<0.0001	0.79
assay	GSH	62.14 (2.34)	45.17 (2.70)	0.0003	38.78 (3.26)	<0.0001	45.66 (1.77)	0.0005	47.64 (1.15)	0.002	40.45 (4.04)	<0.0001	46.37 (1.94)	0.0009	9.33	<0.0001	0.64
CE-ICP-MS	Total iron	1995 (121.1)	2397 (229.5)	0.92	-	-	3541 (228.5)	0.04	3779 (391.1)	0.019	-	-	2947 (581.7)	0.35	4.61	0.012	0.55
	Ferritin-Iron [%]	48.90 (1.33)	45.92 (4.57)	0.99	-	-	70.22 (5.48)	0.03	59.81 (5.85)	0.47	-	-	61.84 (4.31)	0.32	4.67	0.012	0.55
	Fe^{2+}/Fe^{3+}	0.68 (0.05)	1.65 (0.10)	<0.0001	-	-	1.68 (0.06)	<0.0001	1.73 (0.11)	<0.0001	-	-	1.65 (0.12)	<0.0001	23.07	<0.0001	0.86
Immunofluorescence	%NCOA4 in CD11b cells	6.65 (0.87)	-	-	-	-	37.71 (2.75)	<0.0001	-	-	-	-	30.31 (2.35)	<0.0001	51.52	<0.0001	0.89
	nºNCOA4 (30 k)	216 (10.68)	-	-	-	-	699.6 (48.92)	<0.0001	-	-	-	-	-	-	43.81	<0.0001	0.86
	nºNCOA4 (40 k)						734.4 (47.62)	<0.0001									
	nºFerritin (30 k)	42.5 (6.68)	-	-	-	-	684.1 (24.29)	<0.0001	-	-	-	-	-	-	357.4	<0.0001	0.98
	nºFerritin (40 k)						800 (23.11)	<0.0001									
	4-HNE (30 k)	2.53×10^{8} (1.31×10^{7})	-	-	-	-	4.26×10^{8} (2.07×10^{7})	<0.0001	-	-	-	-	-	-	37.32	<0.0001	0.84
	4-HNE (40 k)						5.00×10^{8} (2.30×10^{7})	<0.0001									

m = mean; sem = standard error of the mean; p = p value
